# Ultrafast Interfacial Engineering for Quantifiable Control of Asymmetric Configurations in Nanostructured Janus Membranes

**DOI:** 10.1002/advs.74865

**Published:** 2026-03-16

**Authors:** Wenqing Zhang, Jinhui Xu, Bo Li, Yao Li, Yue Zhang, Zhishu Tang, Huaxu Zhu, Jingwei Hou

**Affiliations:** ^1^ Jiangsu Collaborative Innovation Center of Chinese Medicinal Resources Industrialization, Jiangsu Research Center of Botanical Medicine Refinement Engineering Nanjing University of Chinese Medicine Nanjing China; ^2^ School of Chinese Materia Medica Beijing University of Chinese Medicine Beijing China; ^3^ School of Chemical Engineering The University of Queensland Brisbane Queensland Australia

**Keywords:** Janus membranes, micro‐nano structures, oil‐water separation, tunable wettability, ultrafast fabrication

## Abstract

Janus membranes (JMs) promise advanced liquid separations, but fabrication speed, substrate universality, and precise configurational control remain challenging. Here, we report an ultrafast and universal interfacial engineering strategy to construct JMs with desirable micro‐nano structures. Founded on a rapidly formed tannic acid/polyethyleneimine (TA/PEI) platform, the approach utilizes highly reactive stearoyl chloride (SC) for minute‐scale hydrophobic modification. Crucially, a novel physicochemical control principle is revealed. Specifically, interfacial wettability and tension are modulated at the molecular level by phytic acid (PA). Through this modulation, the membrane's physical immersion depth is governed to provide unprecedented, quantifiable control over hydrophilic layer thickness during liquid‐liquid interface modification. This enables tailored JMs exhibiting pronounced asymmetric super‐wettability (water contact angle (WCA) difference >157°) and tunable unidirectional liquid transport. Demonstrated across diverse metallic and polymeric substrates, the resulting JMs show remarkable separation performance for challenging mixtures, alongside exceptional stability. This facile and controllable strategy overcomes previous limitations in speed, universality, and regulation of asymmetric configurations, offering a versatile platform for designing nano‐engineered functional materials for sophisticated liquid separation applications.

## Graphical abstract

Founded on a rapidly formed nanostructured tannic acid/polyethyleneimine (TA/PEI) platform, an ultrafast (∼5 min), universal interfacial engineering strategy utilizing stearoyl chloride (SC) and Phytic acid (PA) enables precise fabrication of Janus membranes across diverse substrates. PA modulates interfacial wettability to tune hydrophilic layer thickness, yielding asymmetric super‐wettable membranes that achieve efficient, on‐demand oil‐water separation.

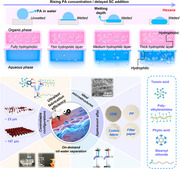

## 1 Introduction

A combination of hydrophilic and hydrophobic modules yields unique liquid transport effects in nature, such as fog collection of beetle's back and water removal of cicada's wing [1, 2]. Inspired by these phenomena, materials with heterogeneous wettability were developed and widely used in oil‐water separation, fog collection, moisture management, and wound healing [3, 4, 5, 6, 7]. As a high‐efficiency and easy‐operation technology, membrane‐based Janus materials with asymmetric wettability show promising prospects in the field of oil‐water separation [8, 9, 10]. Generally, the hydrophilic side of Janus membranes (JMs) allows water to pass through while repelling oil, whereas the hydrophobic side permits oil to pass through while rejecting water [11, 12, 13].

Recognizing the crucial role of Laplace forces, recent research has focused on precisely controlling the thickness of hydrophilic/hydrophobic layers in JMs to achieve distinct liquid transport behaviors [14]. For instance, various studies have successfully engineered JMs with a thin hydrophobic/thick hydrophilic structure to collect trace water from oil in the direction of the hydrophobic side to hydrophilic side, or the inverse configuration to recover trace oil from water in reverse direction [15, 16, 17]. While these specialized designs are highly effective for their intended purpose, they often result in membranes with a fixed, singular functionality (either for water or oil transport). This presents an opportunity to develop a more versatile approach for complex oil‐water separation tasks. Therefore, creating an ideal strategy to fabricate JMs with a controllable‐thickness hydrophilic layer is highly attractive, as it would enable on‐demand oil‐water separation tailored to specific and varying conditions.

Generally, JMs are commonly fabricated via three primary principles: bi‐layer compositing, physical gradient modifying, and interface templating. However, each route possesses inherent limitations. Bi‐layer compositing, while effective for thickness control, can be hindered by delamination issues and the need for specialized equipment [18]. Physical gradient methods, while capable of generating asymmetric properties across the membrane, are frequently restricted by the attenuation of mass or energy (e.g., light transmittance) through the substrate materials [19]. Interface templating, such as single‐side modification at the air‐liquid or liquid‐liquid interface, was wildly utilized for the fabrication of JMs due to its simplicity. However, they struggle to simultaneously achieve high preparation efficiency, substrate universality, tunable thickness of hydrophilic layer, and ideal micro‐nano structures. For instance, single‐sided liquid etching allows efficient control of hydrophilic layer thickness but is typically confined to copper substrate [20, 21]. Although universal coating strategies, such as single‐side polydopamine (PDA) deposition [22, 23, 24, 25] and surfactant‐induced wetting, have successfully extended JMs configurations to diverse substrates, including various fabrics [13], polymeric membranes [26, 27], and filter systems, these methodologies are often constrained by prolonged fabrication kinetics or lack of the hierarchical micro‐nanostructures necessary for robust super‐wettability [28]. Moreover, while advanced techniques such as femtosecond laser microfabrication offer exceptional durability and mode‐switching capabilities, their broad application is hindered by the requirement for specialized and costly equipment [29]. Consequently, there remains a critical need for a universal and efficient platform that provides quantifiable control over both hydrophilic and oleophilic layer thicknesses, thereby enabling the tailored design of JMs for high‐performance, on‐demand separation.

Herein, a universal and ultrafast interfacial engineering strategy is introduced for fabricating JMs with precisely tunable structures at the interface of hexane and water, thereby overcoming the limitations of fabrication kinetics and structural precision. The strategy is founded on a versatile tannic acid/polyethyleneimine (TA/PEI) nanostructured platform, which facilitates rapid hydrophobic modification in minutes using the highly reactive stearoyl chloride (SC) [30, 31]. Central to our report is the discovery of a novel physicochemical control principle: interfacial wettability and tension are actively modulated at the molecular level through the strategic addition of phytic acid (PA) to the aqueous phase. This modulation governs the membrane's physical immersion depth at the liquid‐liquid interface, thereby affording unprecedented, quantifiable control over the hydrophilic layer thickness. This structural precision enables the on‐demand fabrication of JMs with programmable unidirectional transport properties for either water or oil. Demonstrated on diverse substrates—including stainless steel mesh, cotton fabric, PP membranes, and filter paper—this simple yet robust method yields membranes with remarkable performance in separating challenging industrial oil/water mixtures and complex essential oils. Moreover, the membranes exhibit exceptional durability, maintaining stable asymmetric super‐wettability under rigorous mechanical, chemical, and thermal stresses.

## 2 Results and Discussion

### 2.1 Characterization and Optimization of TP and TP‐SC SSM

**SCHEME 1:**
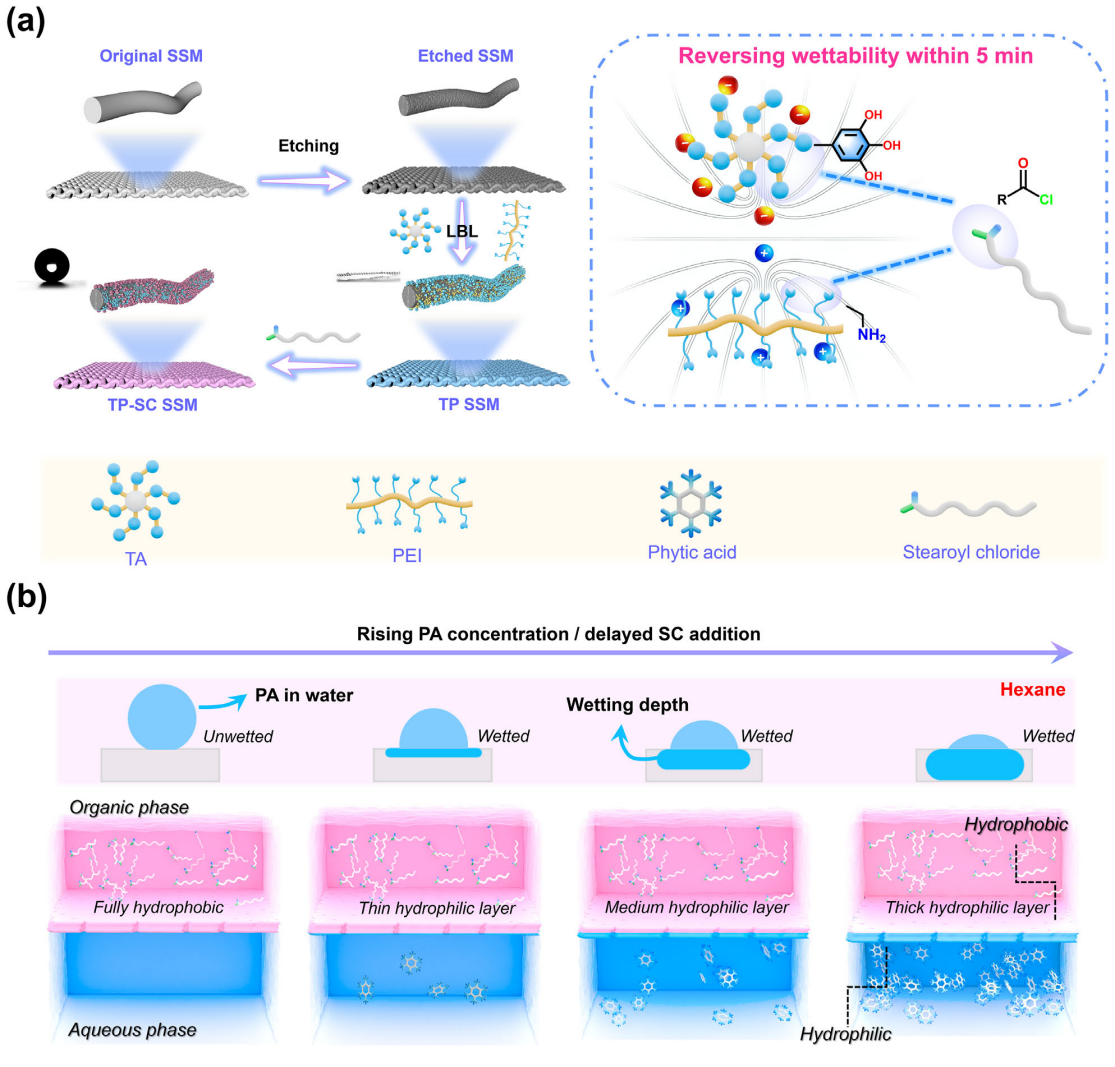
Schematic illustration of (a) TA‐PEI (TP)‐based modification and (b) ultrafast interfacial engineering for quantifiable control of asymmetric configurations in JMs by water protection strategy.

**FIGURE 1:**
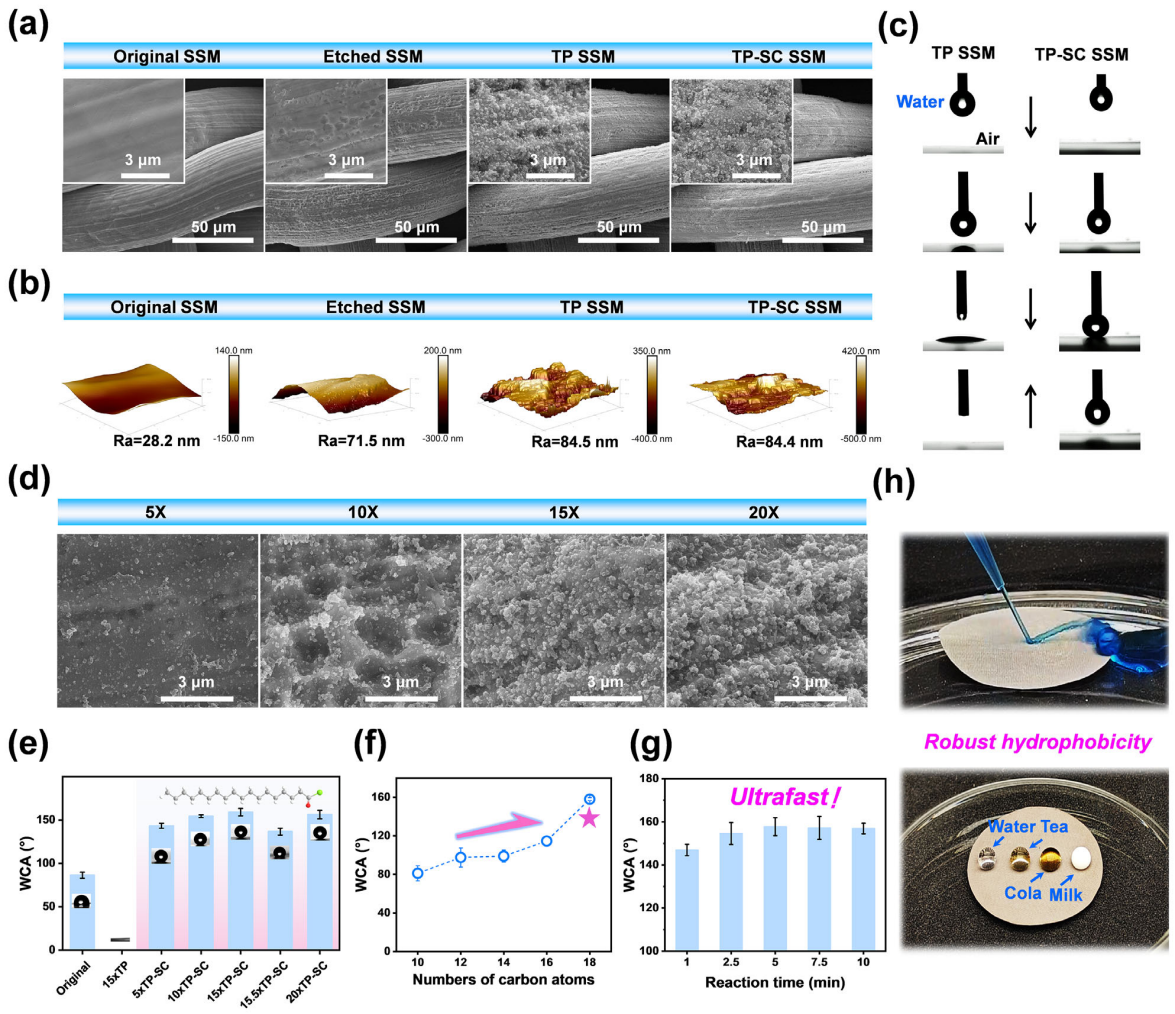
(a) Morphological and (b) 3D topographic images (X: 3 µm; Y: 3 µm) of original, etched, TP modified, and TP‐SC modified SSM. (c) Wettability of TP and TP‐SC SSM. (d,e) The influence of the LBL layer on surface morphology and WCA of TP‐SC SSM. (f) The influence of alkyl acyl chlorides with different lengths of hydrophobic chains on hydrophobic performance. (g) The impact of SC modification time on the WCA of TP‐SC SSM. (h) Optical images of the robust hydrophobic TP‐SC SSM.

The process of TP‐based modification was presented in Scheme 1. Given that the JMs are constructed upon an innovative TP‐based platform, a systematic optimization of this platform's microstructure and wettability was performed first. Compared to the smooth and intact surface of the original SSM, the SSM after treatment with ferric chloride solution showed unevenly sized concave structures and markedly different surface topography (Figure 1a,b), indicating etching was successfully performed. The introduced Fe^3+^ in the TA solution (∼0.05 mM) from the etching step not only strengthen the interaction between modifiers and substrates but also promote the formation of larger TP assembled aggregates on membranes (Figures S1–S3). After TP deposition, the TP self‐assembled particles were evenly distributed on etched SSM, transforming the SSM's wettability into superhydrophilicity. To further investigate the impact of Fe^3+^ in TA solution on TP SSM specifically, different amounts of Fe^3+^ were added into the TA solution before the LBL process. At low Fe^3+^ concentration, almost no micro‐nano structures form, corresponding to a lack of super‐wettability. As Fe^3+^ concentration increases, aggregates grow larger, accompanied by the emergence of superwetting phenomena (Figures S4–S6).

The formed TP‐based reactive surface enabled the straightforward transformation of the hydrophilic TP SSM into a hydrophobic membrane via reaction with SC in hexane (Figure 1c). The maintained microstructure creates a cushion of air between the droplet and the contact surface, also called a Cassie State, facilitating the formation of super‐hydrophobicity [32, 33]. Furthermore, the LBL and SC modifications conformally coated the steel wires without blocking the mesh pores (Figure 1a, Figure S7), preserving the inherent liquid transport channel of the SSM substrate. When the number of TP layers increases from 5 to 15, the WCA of TP SSM remained zero, and TP‐SC SSM monotonously raised from 143.4° to 159.1° (Figure 1e). The increased hydrophobicity was owing to more deposited TP particles on SSM surface, facilitating the formation of micro‐nano structures conducive to super‐hydrophilicity (Figure 1d). As TP layer continuously increased to 20, the WCA of 20× TP‐SC SSM seems to be similar to WCA of 15× TP‐SC SSM, implying the plateau of WCA of TP‐SC SSM with TP‐based microstructure was approximately 160°. Furthermore, the nature of the outermost layer significantly affects the efficiency of the subsequent hydrophobic grafting. A comparison revealed that TP‐SC SSM with PEI as the outermost layer (15×) exhibited higher hydrophobicity than when TA was the outer layer (15.5×). X‐ray photoelectron spectroscopy (XPS) confirmed more efficient SC grafting onto the PEI layer, evidenced by a more substantial decrease in the O/C ratio, likely due to the chelation interaction between Fe^3^^+^ and phenolic hydroxyl groups in the TA layer reducing the number of active sites for SC modification (Figure S8). Thus, 15 LBL cycles terminating with PEI were selected as the optimal condition.

The influence of the alkyl chloride chain length on the hydrophobic modification was investigated. The WCA gradually rises with increasing chain length, especially in SC (Figure 1f). XPS analysis confirmed both the highest carbon content and the absence of residual chlorine for the SC‐modified membrane, indicating that superior performance stems from the combination of the longest chain and efficient reaction (Figure S9). Key advantages of this acyl chloride chemistry include its rapid kinetics and no requirement for additional catalysts. The transformation from super‐hydrophilic TP SSM to super‐hydrophobic TP‐SC SSM occurred within just 5 min (Figure 1g). The resulting TP‐SC SSM demonstrated exceptional and robust hydrophobicity, immediately repelling a dyed water jet and maintaining high contact angles (>150°) for various liquids, including water, tea, cola, and milk (Figure 1h). Meanwhile, the scalability of TP and TP‐SC membranes has been demonstrated in Figure S10, in which substrates with a diameter of 23 cm can be easily modified.

### 2.2 Fabrication and Characterization of JMs

**FIGURE 2:**
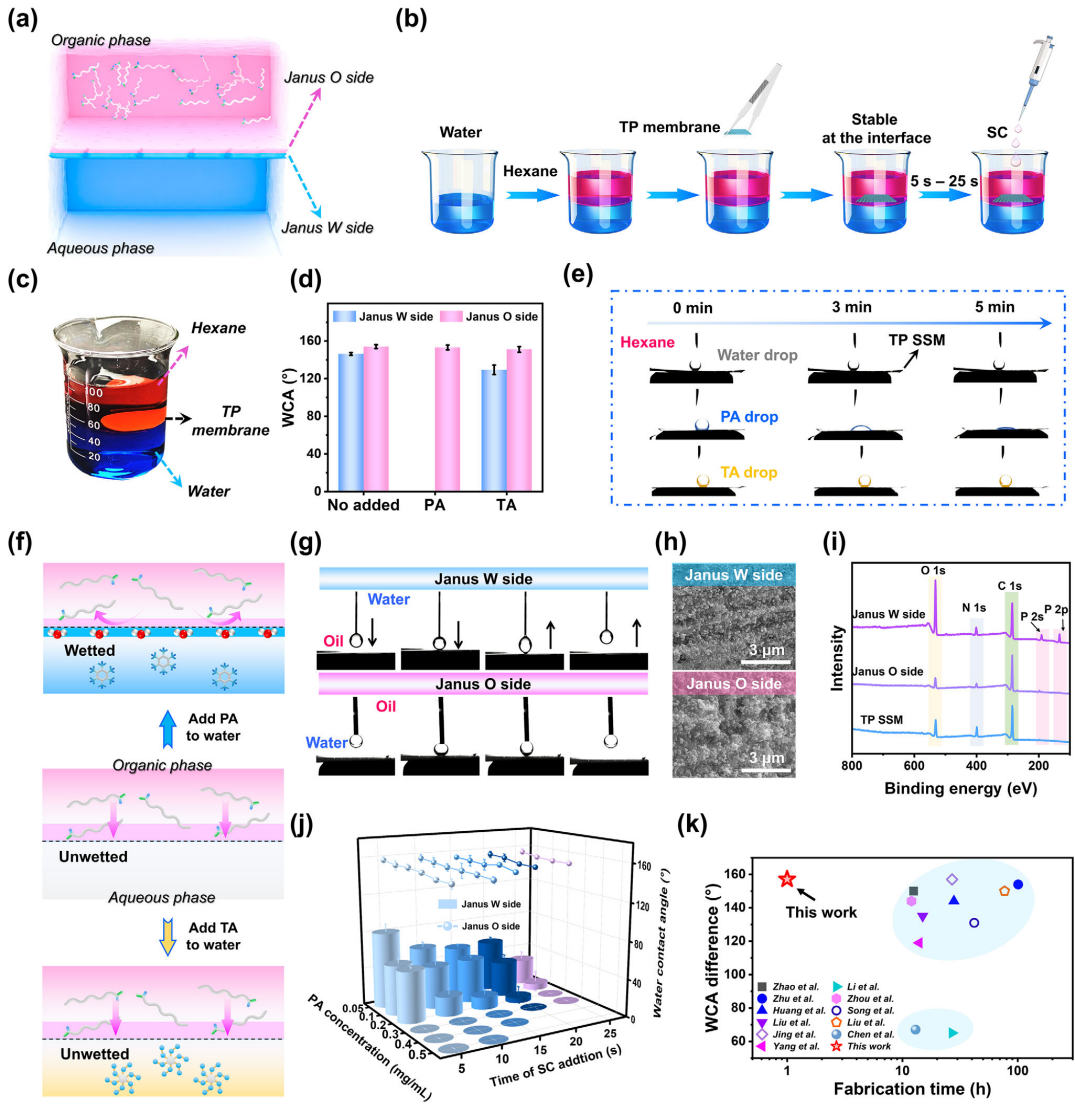
(a) Schematic diagram for fabricating JMs. The pink portion refers to hydrophobic layer (Janus O side) of JMs, while the blue portion represents hydrophilic layer (Janus W side) of JMs. (b) The process of JMs fabrication. (c) The state of TP SSM in water‐hexane interface. Hexane is stained with Oil Red O, while water is stained with Methylene Blue. (d) The influence of solutes in water on the wettability of JMs. (e) The influence of solutes in water on UOWCA of TP SSM. (f) The underlying mechanism of water protection strategy in the process of JMs fabrication. (g) The excellent under‐water oleophobic of Janus W side and under‐oil hydrophobic of Janus O side. (h) SEM results of Janus W side and Janus O side of JMs. (i) XPS results of TP SSM, Janus W side, and Janus O side. (j) The impact of PA concentration in aqueous solution and the time of SC addition on the WCA of JMs. (k) Comparison of this study with reported studies in preparation time of JMs and WCA difference between Janus W side and Janus O side [11, 15, 17, 20, 34, 35, 36, 37, 38, 39, 40].

The fabrication of JMs was attempted using a water protection strategy at the interface between water and hexane, where the TP membranes can stably exist (Figure 2a–c). The hypothesis was that SC, soluble only in hexane and hydrolyzing in water, would selectively and quickly reverse the wettability (5 min) of the super‐hydrophilic membranes residing in the hexane phase (Janus O side), while the portion immersed in the aqueous phase (Janus W side) is protected by water and exhibits hydrophilicity (Figure 2a). However, direct application of this strategy using pure water yielded a fully hydrophobic membrane (Figure 2d), with both sides exhibiting high WCA, indicating a failure to construct asymmetric wettability. The underlying reason was identified as the TP SSM's extremely high under‐oil hydrophobicity (Figure S11), preventing the membrane from being wetted by the water phase.

To alter the under‐hydrophobicity of TP membranes preventing Janus formation, we explored additives for the aqueous phase. Exploration revealed that phytic acid (PA), containing six phosphonic acid groups known for conferring hydrophilicity [41, 42], dramatically lowered the UOWCA of the TP SSM (Figure 2e). This provided the crucial under‐oil hydrophilicity of TP SSM needed for the water protection strategy (Figure 2f). Consequently, incorporating PA into the aqueous phase during the liquid‐liquid modification successfully produced the desired Janus architecture. The Janus O side retained its SC‐induced super‐hydrophobicity and under‐oil water repellency. Simultaneously, the Janus W side, wetted and protected by PA solution, achieved super‐hydrophilicity and under‐water oleophobic (Figure 2d,g, Figure S12). This outcome contrasts sharply with attempts using TA, which failed to change the under‐oil hydrophobicity of TP SSM, yielding a membrane with homogeneous wettability. To further investigate the mechanism of PA‐induced UOWCA modulation, theoretical analysis and molecular dynamic (MD) simulations were carried out.

(1) $$\cos\theta =\frac{\gamma_{hm-}\gamma_{wm}}{\gamma_{hw}}$$

where γ_*hm*_ is the interfacial tension at the hexane/membrane interface, γ_*wm*_ is the interface tension at the water/membrane interface, γ_*hw*_ is the interface tension at the hexane/water interface, and θ is the UOWCA. γ_*hm*_ must be larger than γ_*wm*_ for an underoil hydrophilic surface (θ≤ 90°), which means the membrane has a stronger affinity to PA solution than hexane [43]. The interaction energy between membrane‐hexane and membrane‐PA solution was analyzed by MD simulation. As shown in Figure S14, the interaction energy of membrane to PA solution (−67,513 kJ mol^−1^) was significantly stronger than membrane to hexane (−22,449 kJ mol^−1^), especially electrostatic energy. Therefore, though the TP membrane was immersed in hexane, the PA drop can still wet the membrane.

SEM revealed that the Janus W and Janus O sides preserved the nanostructured morphologies characteristic of the parent TP SSM and TP‐SC SSM, respectively, providing the structural basis for asymmetric super‐wettability (Figure 2h). Meanwhile, XPS characterization was employed to accurately investigate the chemical composition of the two sides of JMs (Figure 2i, Figure S15). Compared to the TP SSM, the peaks of the P element appear and the intensity of O 1s remarkably increased in the Janus W side, indicating PA was successfully adsorbed in the membrane. In Janus O side, the strength of C 1s was significantly enhanced due to the introduction of SC with a long hydrophobic chain.

As PA concentration increased, SC addition was delayed, the Janus W side transitioned toward super‐hydrophilicity (WCA ≈ 0°), while the Janus O side remained robustly super‐hydrophobic, yielding a substantial maximum WCA difference of 157.1° (Figure 2j). Critically, this strategy offers dramatic improvements in efficiency, reducing fabrication time by nearly an order of magnitude (from multiple hours to under one hour) while simultaneously achieving adjustable and significant asymmetric wettability superior to previous methods (Figure 2k). This combination of rapidity, tunability, and high performance provides a strong foundation for expanding JMs applications.

### 2.3 Regulation of JM's Asymmetric Configurations

**FIGURE 3:**
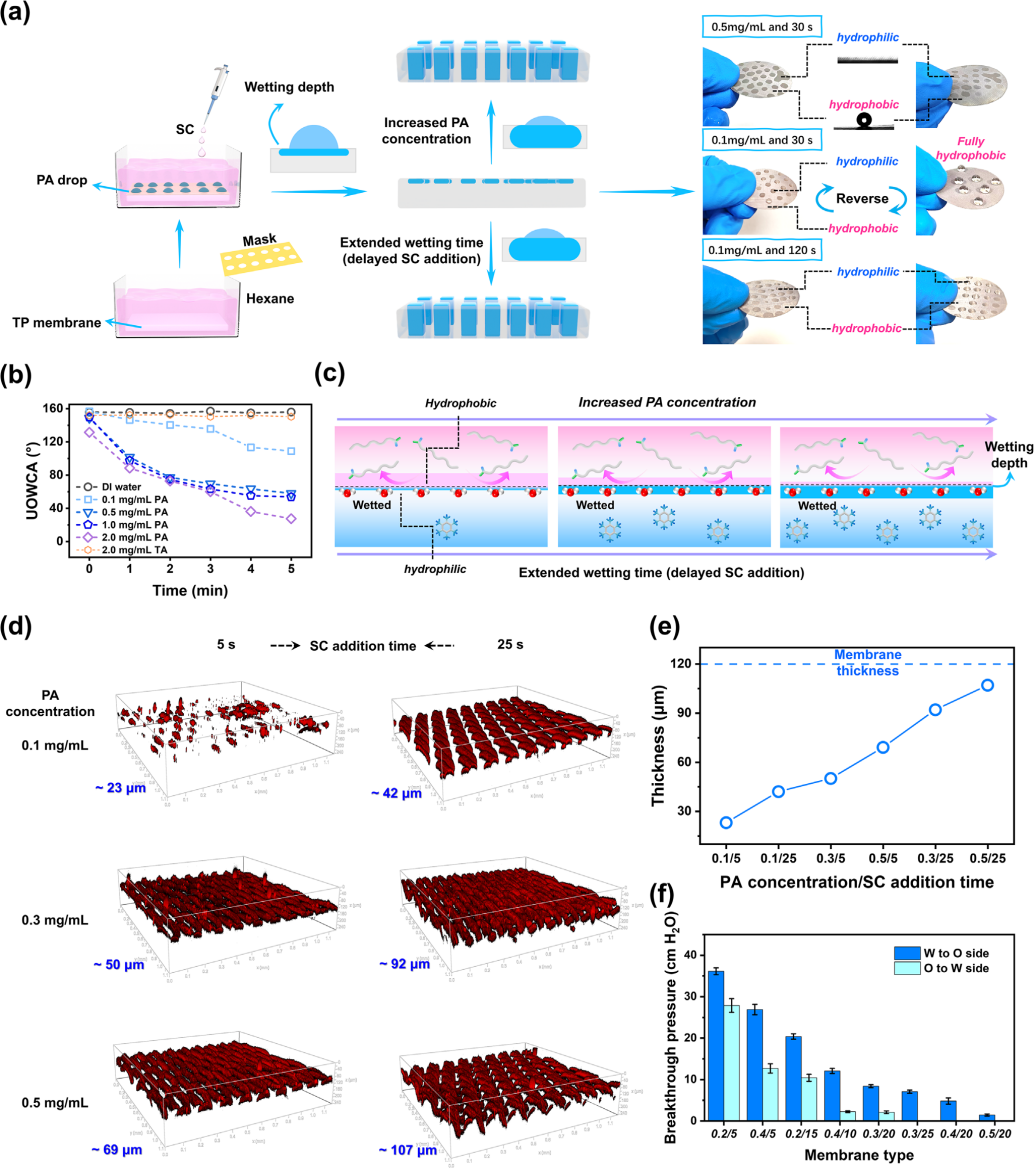
(a) Mask strategy for the fabrication of membranes with distinctive heterogeneous wettable surfaces. (b) The influence of solutes in water on the UOWCA of JMs. (c) The underlying mechanism of the controllable thickness of hydrophilic layer in JMs. (d) CLSM results of JMs fabricated at different PA concentrations and SC addition times. The number in the lower left corner represents the thickness of the fluorescent layer (X: 1.2 mm; Y: 1.2 mm; Z: 250 µm). (e) The thickness of the hydrophilic layer based on CLSM data. (f) The water breakthrough pressure of JMs fabricated at different PA concentrations and SC addition time.

Precise control of the asymmetric configurations in Janus materials has garnered significant attention across numerous fields, including oil‐water separation, wetting control, and membrane distillation [7, 18, 21]. This is primarily attributed to the different thickness of hydrophilic layers, which can impart distinct liquid transport properties to the substrates. The key insight in this work was leveraging the PA concentration and wetting time (SC addition time) to precisely manage the membrane's under‐oil wettability. Figure 3b shows that increasing PA concentration and wetting time significantly decreases the UOWCA of the TP SSM. This reduction dictates the wetting range and depth, thereby defining the extent of water protection crucial for regulating the hydrophilic layer thickness.

To visualize this, a mask strategy was used to create heterogeneous wettability (Figure 3a). Low PA concentrations and short SC addition times resulted in shallow wetting and unilateral hydrophobicity. Conversely, increasing PA or wetting time induced deeper wetting, allowing the hydrophilic region to penetrate the membrane. This regulation of water protection depth applies similarly to the hexane‐water interface (Figure 3c). The UOWCA decrease driven by higher PA concentrations and prolonged wetting times directly governs the wetting depth (hydrophilic layer thickness). This wetting process is arrested by the rapid formation of the TP‐SC layer, which exhibits strong under‐oil hydrophobicity independent of solute concentration and wetting time (Figure S17).

The quantifiable control over JMs’ asymmetric configuration was verified by CLSM. The thickness of the hydrophilic layer (red fluorescence) systematically increased with both higher PA concentrations and delayed SC addition (Figure 3d,e, Figures S18 and S19). For example, at a 5 s SC addition time, increasing PA concentration from 0.1 to 0.5 mg mL^−1^ thickened the layer from ∼23 to ∼69 µm. Changes in JM configuration significantly affect the water breakthrough pressure of JMs (Figure 3f). At lower PA concentrations and shorter SC addition times, a thick hydrophobic layer results in higher breakthrough pressure regardless of liquid transport direction. As the hydrophobic layer thins and the hydrophilic layer thickens, a decreasing trend in breakthrough pressure is observed. When the configuration shifts to a thin hydrophobic layer and thick hydrophilic layer, spontaneous water transport occurs from the hydrophobic to hydrophilic side (Figure S20), reducing breakthrough pressure to zero. Conversely, the W‐to‐O direction can still withstand significant hydrostatic pressure; for example, a 7.1 cm water column was blocked by JMs prepared with 0.3 mg mL^−1^ PA and 25 s SC addition time.

### 2.4 On‐Demand Oil–water Separation by JMs

**FIGURE 4:**
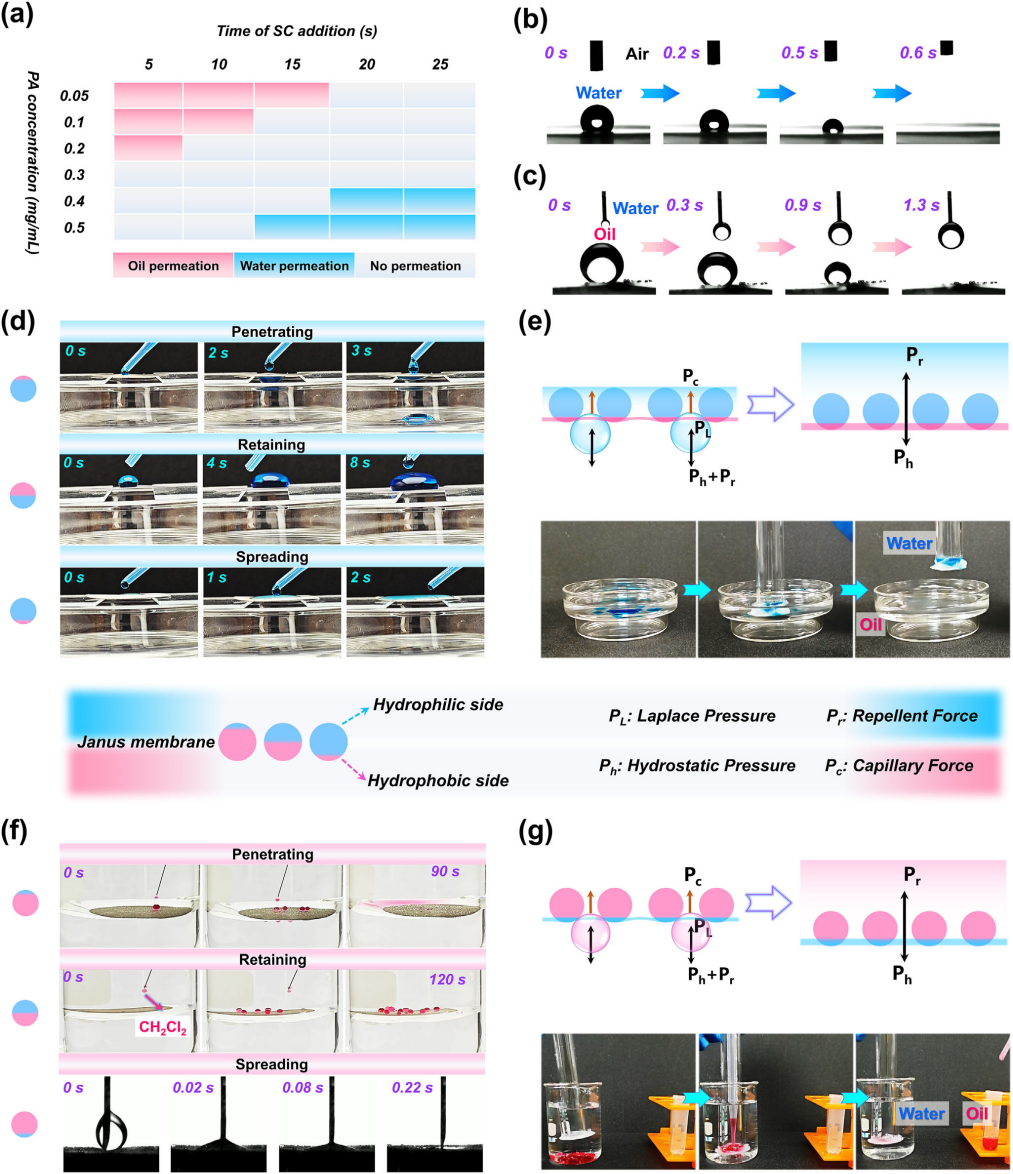
(a) Summary of spontaneous liquid transport. The spontaneous (b) water or (c) oil transport of JMs with different thicknesses of hydrophilic layer. The influence of hydrophilic layer thickness and orientation of JMs on (d) water and (f) oil transport. The mechanism and application of unidirectional (e) water and (g) oil transport. Water was stained by methyl blue, and oil was stained with Oil Red O.

Generally, the hydrophobic side in JMs allows oil to pass through and retaining water, while the hydrophilic surface permits water to pass through but blocks oil [44]. Manipulating the configuration of JMs endows JMs with distinct water/oil transport behavior (Figure 4a, Figures S21 and S22). Under high PA concentration and delayed SC addition, JMs with thick hydrophilic/thin hydrophobic layers can be fabricated to realize spontaneous water transport. Once the water droplet was dropped on the Janus O side of JMs with a thin hydrophobic layer/thick hydrophilic layer, it rapidly permeates from the hydrophobic side to the hydrophilic side (Figure 4b,d, Movie S2). However, a water droplet cannot penetrate through the membrane in the direction from Janus W side to the oil side or in a JM with thicker hydrophobic layer.

At low PA concentration and short SC addition time, thick hydrophobic/thin hydrophilic JMs can be prepared to achieve spontaneous oil transport. (Figure 4c,f, Movie S3). Conversely, when oil was dropped on the Janus W side of JMs with comparable thickness of hydrophilic and hydrophobic layers or on the Janus O side, no oil permeation happened.

To directly separate the trace phase from the bulk phase, a JMs with a thin lyophobic/thick lyophilic layer was used. When the Janus O side contact with water, the sum of *P_L_* (Laplace force) and *P_c_* (Capillary force) is greater than *P_h_* (Hydrostatic Pressure) and *P_r_* (Repellent Force), effectively drawing water into the glass tube. Furthermore, the hydrophobic side's *P_r_* prevents water permeation in the direction of W side to the O side, avoiding water leakage when lifting the glass tube (Figure 4e). Similarly, JMs composed of thick hydrophobic layer/thin hydrophilic layer achieved trace oil recovery from water (Figure 4g).

**FIGURE 5:**
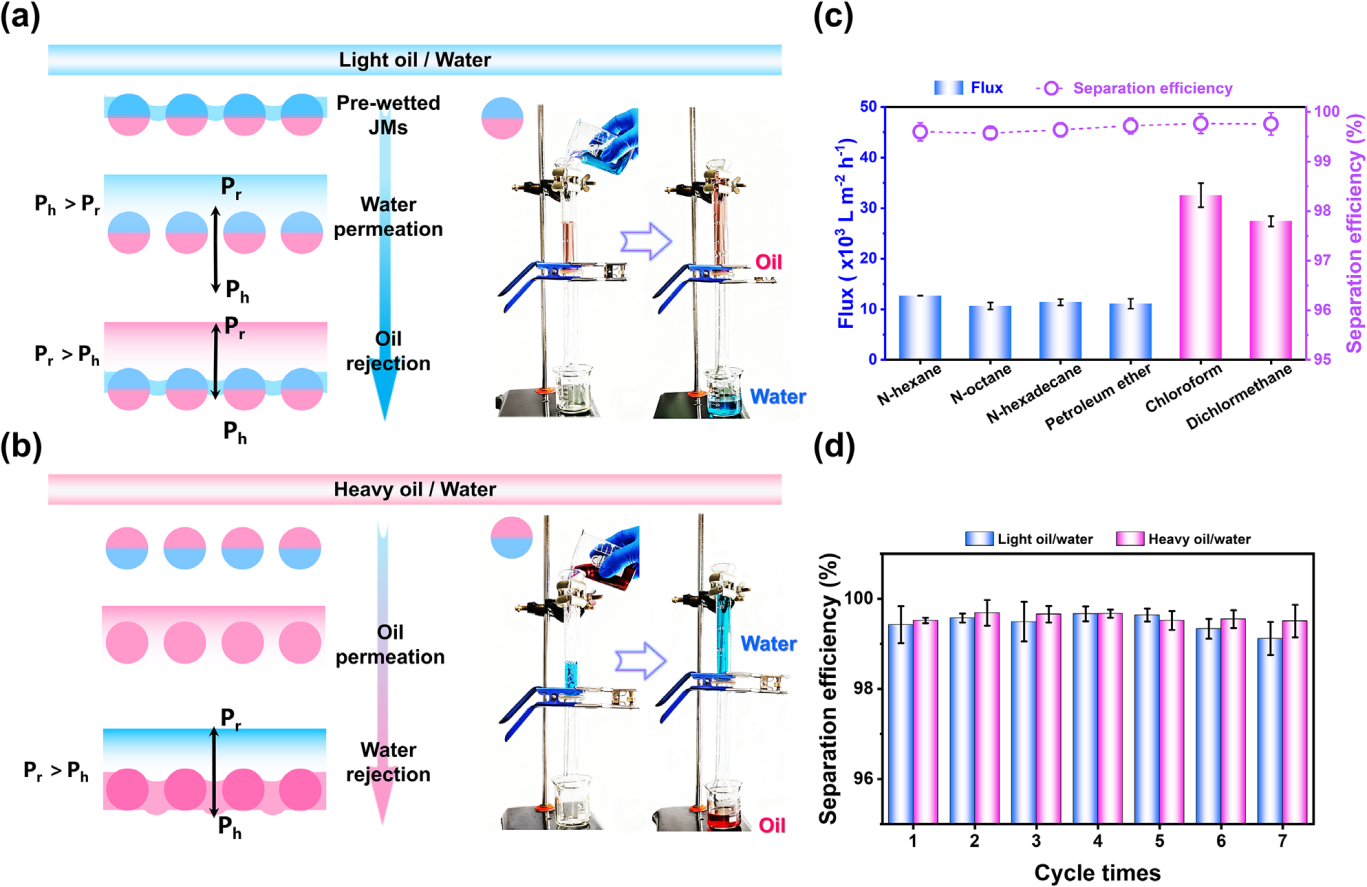
The mechanism and application of JMs in (a) light and (b) heavy oil–water mixtures separation. (c) The flux and separation efficiency of JMs on different immiscible oil‐water mixtures. (d) The reusability of JMs for light oil–water and heavy oil–water separation.

The excellent underwater oleophobic properties of the Janus W side and the hydrophobic properties of the Janus O side also endow JMs with outstanding separation capabilities for oil‐water mixtures with comparable proportions. By adjusting the orientation of the JMs, the denser phase in the mixture can pass through, while another phase is retained (Figure 5a,b). For light oil/water mixtures, pre‐wetted JMs can initially prevent oil permeation. Subsequently, water breaks through the hydrophobic side of the JMs once *P_h_* exceeded *P_r_* as the liquid level rises and oil is ultimately rejected. For heavy oil/water mixtures, liquid permeation happened once oil reached JMs since both the Janus W and O sides exhibit oleophilic properties in air and terminated when water contacted. The water flux of JMs exceeds 10 000 L m^−2^ h^−1,^ and oil flux is around 30 000 L m^−2^ h^−1^. Benefiting from the excellent underwater oleophobic properties of the Janus W side and the hydrophobic properties of the Janus O side, the separation efficiency exceeds 99.5% (Figure 5c). Meanwhile, the excellent reusability of JMs has been confirmed through seven cycles of light or heavy oil/water separation, with separation efficiencies exceeding 99% in each cycle (Figure 5d).

The versatility of the tunable JM platform was further highlighted in the challenging separation of plant‐derived essential oils. Unlike typical immiscible solvents, these complex mixtures—often containing polar, high‐viscosity terpenoids—tend to emulsify and exhibit problematic wall adhesion [45, 46, 47], rendering conventional bulk phase removal ineffective (Figure S23). Our strategy overcomes this by enabling direct recovery of the essential oil phase through precisely tailored JMs designed for unidirectional oil transport (Figure S24). These results confirm that the TP‐based modification allows the fabrication of JMs suited for diverse and demanding oil‐water separation tasks.

### 2.5 Stability of JMs

To examine the stability of TP‐based JMs, sonicating, tape peeling, chemical treatment, thermal incubation, and water flushing tests were carried out. Excellent mechanical stability was confirmed: the asymmetric super‐wettability (Janus O side WCA > 155°, Janus W side underwater oleophobicity) remained intact after 25 min of sonication and 50 cycles of tape peeling, indicating strong adhesion of the TP‐based microstructure to the substrate (Figure S25a,b). Compared to chemically sensitive structures like Cu(OH)_2_ nano‐needles, JMs in this study exhibited superior chemical tolerance, maintaining stable wettability after 2 h of immersion in corrosive solutions at pH 3 and pH 11 (Figure S25c). Furthermore, the membranes demonstrated good thermal stability relevant to applications like membrane distillation [48, 49, 50]. Exposure to water at 60°C for 2 h caused only a minor decrease in the Janus O side WCA (from 154.8° to 148.7°) while the Janus W side wettability was unaffected (Figure S25d).

The stability of JMs under dynamic flow conditions is critical for practical applications (Figure S26a). To evaluate this, the Janus W side was subjected to a rigorous continuous flushing test at a high flow rate of 100 mL min^−1^. As shown in Figure S26b, even after 30 min of high‐velocity flushing, the surface maintained stable underwater super‐oleophobicity. Crucially, SEM observations confirm that the micro‐nano morphology remained intact (Figure S26c). Furthermore, XPS analysis (Figure S26d) reveals that the characteristic P 2p signal intensity remained virtually unchanged, indicating that the PA molecules are firmly anchored to the substrate and do not undergo desorption under hydraulic shear. This robustness against hydraulic stress, combined with the previously demonstrated mechanical and chemical stability, underscores the potential of these JMs for reliable long‐term performance in demanding separation environments.

### 2.6 Universality of TP‐Based Janus Modification Method

**FIGURE 6:**
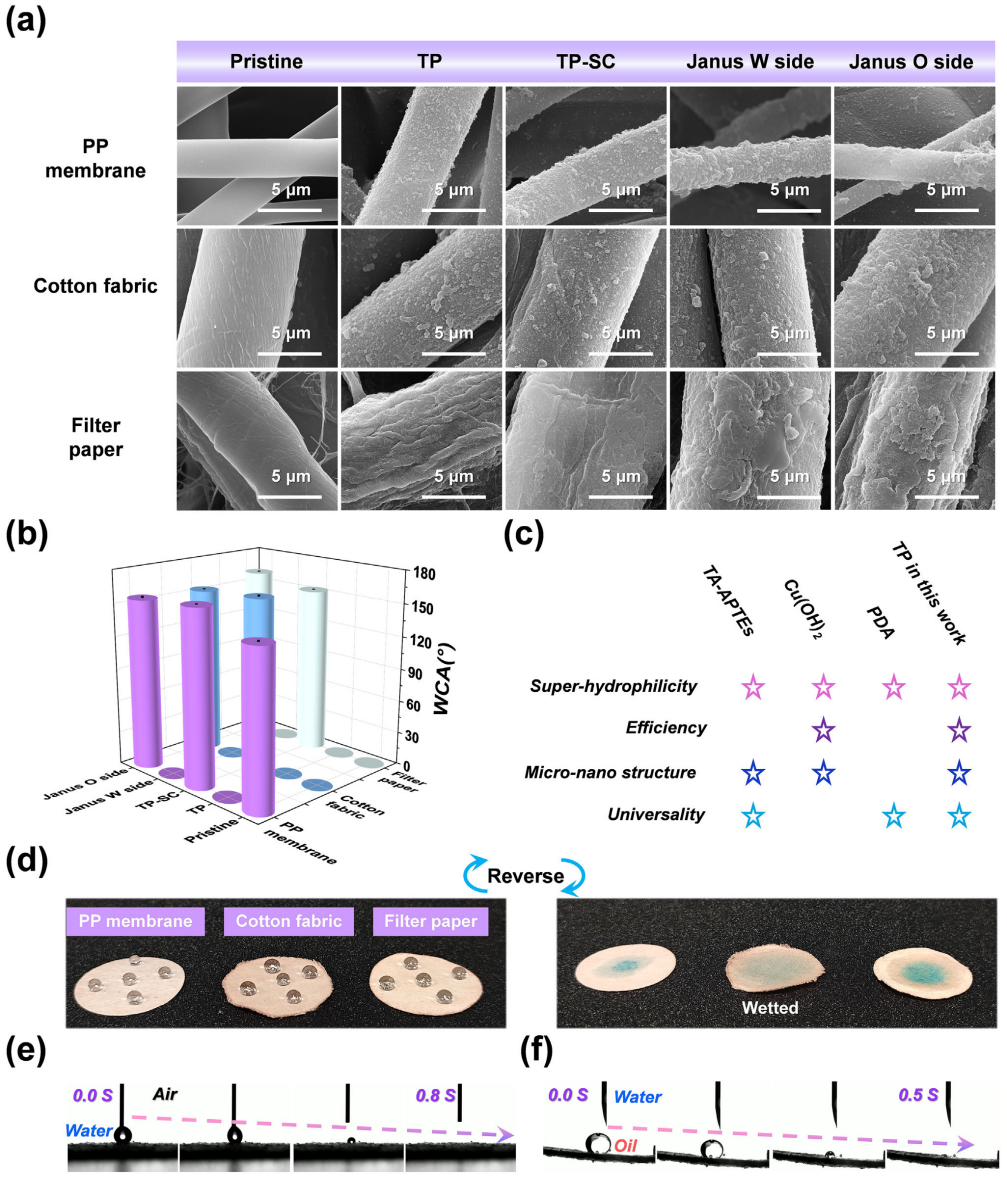
(a) SEM results of pristine, TP, and JMs based on PP membrane, cotton fabric, and filter paper, respectively. (b) The WCA change of different substrates during TP‐based Janus modification. (c) Superiorities of TP‐based LBL modification in this study [15, 20, 24, 51, 54]. (d) Asymmetric wettability of JMs constructed by different substrates. (e‐f) The spontaneous water and oil penetration of JMs composed of a thick hydrophilic /thin hydrophobic layer and thin hydrophilic /thick hydrophobic layer, respectively.

Cotton fabric, PP membrane, and filter paper were employed to verify the universality of TP‐based Janus modification in this study. Compared to the smooth surface of the pristine substrate, the TP‐modified membrane surface exhibits an obvious micro‐nano structure (Figure 6a). The characterization of XPS also demonstrated that the TP‐based modification was successfully achieved (Figures S27 and S28). Even PP membrane was rapidly rendered super‐hydrophilic (within ∼30 min) via ethanol pre‐wetting followed by TP deposition, showcasing the method's versatility. Following SC grafting, the WCA of all TP‐SC membranes fabricated from PP membrane, cotton fabric, and filter paper approaches 160°, exhibiting superior hydrophobicity (Figure 6b, Movies S4–S6). Meanwhile, this microstructure persists regardless of subsequent PA or SC modifications, effectively ensuring the persistence of asymmetric superwetting property (Figure 6d).

Compared to established universal modifications like TA‐APTES nanospheres, PDA coating, and Cu(OH)_2_ nanoneedles [51, 52, 53], the TP‐based LBL platform offers a distinct advantage. While methods like Cu(OH)_2_ growth are fast but substrate‐specific. PDA or TA‐APTES offer universality but suffer from slow deposition times. our TP approach uniquely combines rapid fabrication, broad substrate compatibility, and the inherent formation of microstructures beneficial for super‐wettability (Figure 6c). This positions the TP‐based strategy as a highly efficient and versatile platform for creating tailored JMs across a wide range of materials.

Crucially, precise control of asymmetric configurations was achieved on these varied materials by adjusting PA concentration and SC addition time. The unidirectional liquid transport (spontaneous water vs. oil permeation) was also successfully demonstrated, with results using PP membrane illustrating this principle (Figure 6e,f, Movies S7 and S8). JMs with asymmetric wettability can also be used for emulsion separation. To enhance the role of aperture screening in separation, a 0.22 µm PP membrane was employed as a substrate. When Janus W side facing feed oil‐in‐water emulsion, the excellent hydrophilicity and underwater oleophobicity achieved water permeation and oil rejection with separation efficiency over 99%. The flux exceeded 2000 L m^−2^ h^−1^ bar^−1^ during the separation of n‐hexane in water, n‐octane, and n‐hexadecane in water emulsion. Similarly, the superior hydrophobicity of the Janus O side enables oil permeation and water rejection when it facing water‐in oil emulsion. The separation efficiency of the emulsion is more than 99%, and the oil flux exceeds 1000 L m^−2^ h^−1^ bar^−1^ (Figures S29–S31). These results underscore the effectiveness of the TP‐based Janus modification in producing high‐performance membranes suitable for demanding emulsion separation tasks.

## 3 Conclusion

In summary, this work introduces an efficient strategy for fabricating wettability‐tunable JMs with favorable micro‐nano structures. Founded on a rapidly formed TA/PEI platform optimized with trace Fe^3^^+^, it enables minute‐scale hydrophobic modification via SC. Crucially, a novel physicochemical control principle is established. Specifically, interfacial wettability and tension are modulated at the molecular level by PA. Consequently, the membrane's immersion depth is governed, providing quantifiable control over hydrophilic layer thickness during liquid‐liquid interface modification. This allows swift (<1 h) fabrication of JMs featuring tailored asymmetric configurations and tunable unidirectional liquid transport. Demonstrated across diverse substrates, the membranes exhibit remarkable separation performance for challenging oil‐water mixtures and exceptional mechanical, chemical, and thermal robustness. This facile, structure‐controllable strategy overcomes key limitations in speed, universality, and tunability, offering a powerful platform for advanced Janus materials targeting sophisticated, multi‐scenario liquid separation.

## 4 Experimental Section

### 4.1 Materials

Stainless steel mesh (SSM, 1000 mesh size) and cotton fabric were obtained from local markets. Polypropylene (PP) membrane and filter paper were purchased from Haining Delv New Material Technology Co., LTD (Haining, China). FeCl_3_·6H_2_O, ethanol, n‐hexane, petroleum ether, chloroform, and Span‐80 were purchased from Sinopharm Chemical Reagent Co., Ltd. (Shanghai, China). Tween‐80 was obtained from Solarbio Science & Technology Co., Ltd. (Beijing, China). Decanoyl chloride, lauroyl chloride, myristoyl Chloride, palmitoyl chloride, stearoyl chloride (C18), polyethyleneimine (PEI, 70 000 g mol^−1^), n‐hexadecane, dichloromethane, n‐octane, and phytic acid (PA) were purchased from Aladdin Biochemical Technology Co., Ltd. (Shanghai, China). Tannic acid (TA) was purchased from Nantong Feiyu Biological Technology Co., Ltd. (Jiangsu, China). All the chemicals were used directly without further purification.

### 4.2 Fabrication of TP and TP‐SC SSM

SSMs with an area of 7.1 cm^2^ were etched in 20 mL 18% (*w/w*) FeCl_3_ and 0.1 M HCl aqueous solution for five minutes. Subsequently, the etched SSM was gently washed by DI water. TP (TA/PEI) SSM was constructed by layer‐by‐layer (LBL) self‐assembly between TA and PEI. The etched SSMs was immersed in 100 mL 2 mg mL^−1^ TA solution for 30 s and the poorly bonded reactants were removed with deionized (DI) water. Then TA SSM was transferred to 100 mL 2 mg mL^−1^ PEI solution for 30 s. The weakly bonded PEI was also washed away by the water stream. The above steps were repeated 5–20 times to obtain 5×, 10×, 15×, 20× TP SSM. The dried TP SSM was placed in n‐hexane with 5 mg mL^−1^ SC and reacted for 5 min to obtain the TP‐SC SSM.

### 4.3 Fabrication of JMs

30 mL of aqueous solution with different PA concentrations (0.05, 0.1, 0.2, 0.3, 0.4, 0.5 mg mL^−1^) and 30 mL of n‐hexane solution were poured sequentially into a beaker to form a two‐phase solution. TP SSM immersed in the above solution was able to float on the interface of water and n‐hexane. 150 mg of SC was added to the above n‐hexane at 5, 10, 15, 20, and 25 s after TP SSM stabilization. It is important to note that SC should be stored at temperatures above 30°C to maintain its liquid state. The side of the SSM facing water is called the Janus W (water) side, in which positively charged PEI can adsorb the PA in aqueous solution through electrostatic interactions. The side facing the n‐hexane is defined as the Janus O (oil) side, where the amino group on the PEI or the phenolic hydroxyl group on the TA can react with the SC in the organic phase. After 5 min, the membrane was washed with ethanol to remove unreacted reactants and dried at air oven. Specific steps can be found in Figure 3a and Movies S1.

### 4.4 Separation of Immiscible Oil–Water Mixtures

The separation of immiscible oil–water mixtures was performed using JMs with comparable hydrophilic and hydrophobic thickness (PA concentration = 0.3 mg mL^−1^, SC addition time = 15 s). During the oil‐water separation experiment, the feed solution consisting of 25 mL of water and 25 mL of oil was rapidly poured into the separation device. JMs are fixed between two glass tubes with an effective filtration area of 1.77 cm^2^. When separating light oil/water mixtures, the pre‐wetted JMs are oriented with the Janus W side facing upward and the Janus O side facing downward. When separating heavy oil/water mixtures, the JMs are oriented with the Janus O side facing upward and the Janus W side facing downward. All separation occurs under the effect of gravity. The formulas for water flux and separation efficiency are shown below.

(2) $$Flux=\frac{V}{A\times t}$$

(3) $$Separationefficiency=\frac{M_{2}}{M_{1}}\times 100\%$$

where *V* represents volume of filtrate (L), A is the membrane filtration area (1.77 cm^2^) and *t* refers to the filtration time (h). *M*_1_ is the water mass (light oil/water separation) or the oil mass (heavy oil/water separation) before separation, and *M*_2_ is the collected filtrate after filtration.

### 4.5 Separation of Oil/Water Emulsions

To achieve the separation of emulsions containing micron‐sized dispersed phases, a 0.22 µm PP membrane was employed as the substrate to construct a small‐pore JM. When separating water‐in‐oil (W/O) emulsions, the Janus W side of the pre‐wetted JMs faces the feed emulsion. The Janus O side of the pre‐wetted JMs faces the feed emulsion when separating oil‐in‐water (O/W) emulsions. A dead‐end vacuum filtration apparatus with 1.77 cm^2^ of effective filtration area was employed for separating water‐in‐oil emulsions at a negative pressure of 0.015 bar and oil‐in‐water emulsions at the pressure of gravity. The surfactant‐stabilized oil‐in‐water emulsion feed was prepared by adding 1 mL of oil (n‐hexadecane, isooctane, dodecane, or kerosene) and 0.05 g of Tween‐80 in 99 mL of DI water, followed by vigorous mechanical stirring at 1500 rpm for 4 h. The surfactant‐stabilized water‐in‐oil emulsion feed was prepared by adding 2 mL of water to 98 mL of oil containing 0.05 g Span‐80, followed by vigorous mechanical stirring at 1500 rpm for 4 h. The formula's separation efficiency of the emulsion was shown below.

(4) $$Separationefficiency=\left(1-\frac{C_{f}}{C_{p}}\right)\times 100\%$$

where *C*_p_ and *C*_f_ represent the oil content (O/W emulsion separation) or water content (O/W emulsion separation) in pristine emulsion and filtrate, respectively.

### 4.6 Characterization

Scanning electron microscopy (SEM, QUANTA 250 FEG, Thermo Fisher Inc., USA) was used to characterize the morphology of different membranes. Membrane wettability was analyzed via contact angle measurement (ZJ‐7000S, Z. Jia Instrument Equipment Co., Ltd., Shenzhen, China). Specifically, about 2 µL of water was dripped onto the mesh in air to obtain the water contact angle (WCA). 5 µL of oil was dripped onto the mesh in water to analyze the underwater lipophobicity. 5 µL of water was dripped onto the membrane in oil to analyze the underoil hydrophobicity. The elements of the membranes were acquired by X‐ray photoelectron spectroscopy (XPS, AXIS ULTRA DLD, Shimadzu Inc., Japan). Laser scanning confocal microscopy (LSCM, Leica‐STELLARIS 5, Germany) was used to quantify the thickness of JMs' hydrophilic layer stained with rhodamine B. The water content in oil was determined using a Karl Fischer titrator (Byes‐2000, Bangyi Precision Measuring Instruments (Shanghai) Co., Ltd., Shanghai, China). The oil content in water was calculated using chemical oxygen demand, which measured by a LY‐3D type multi‐function water quality tester (Qingdao Luyu Environmental Protection Technology Co., Ltd., China).

## Funding

National Natural Science Foundation of China (Project No. 82274222, 82274107), the Open Project of Chinese Materia Medica First‐Class Discipline of Nanjing University of Chinese Medicine (No. ZYXYL2024‐013), Jiangsu Province Leading Talents Cultivation Project for Traditional Chinese Medicine (SLJ0304), Innovation Research on Interdisciplinary Sciences at Nanjing University of Chinese Medicine (JC202403), and the ability establishment of sustainable use for valuable Chinese medicine resources (2060302).

## Conflicts of Interest

The authors declare no conflicts of interest.

## Supporting information




**Supporting File 1**: advs74865‐sup‐0001‐MovieS1.mp4.

**Supporting File 2**: advs74865‐sup‐0002‐MovieS1.avi.

**Supporting File 3**: advs74865‐sup‐0003‐MovieS3.avi.

**Supporting File 4**: advs74865‐sup‐0004‐MovieS4.mp4.

**Supporting File 5**: advs74865‐sup‐0005‐MovieS5.mp4.

**Supporting File 6**: advs74865‐sup‐0006‐MovieS6.mp4.

**Supporting File 7**: advs74865‐sup‐0007‐MovieS7.avi.

**Supporting File 8**: advs74865‐sup‐0008‐MovieS8.avi.

**Supporting File 9**: advs74865‐sup‐0009‐SuppMat.docx.

## Data Availability

The data that support the findings of this study are available from the corresponding author upon reasonable request.
