# Supplementary material for: Ultrafast Interfacial Engineering for Quantifiable Control of Asymmetric Configurations in Nanostructured Janus Membranes
Source: Adv Sci (Weinh). 2026 Mar 16;13(30):e74865. doi: 10.1002/advs.74865 (PMC13248778; doi:10.1002/advs.74865)
Supplement: Supplementary file 9 — Supporting File 9: advs74865‐sup‐0009‐SuppMat.docx. [file ADVS-13-e74865-s003.docx]

SUPPORTING INFORMATION

Ultrafast Interfacial Engineering for Quantifiable Control of Asymmetric Configurations in Nanostructured Janus Membranes

Wenqing Zhang ^a, b^, Jinhui Xu ^a, b^, Bo Li ^a, b, *^, Yao Li ^a, b^, Yue Zhang ^a, b^, Zhishu Tang ^c^, Huaxu Zhu ^a, b, *^, Jingwei Hou ^d, *^

*^a^ Jiangsu Collaborative Innovation Center of Chinese Medicinal Resources Industrialization, Nanjing University of Chinese Medicine, Nanjing 210023, China*

*^b^ Jiangsu Research Center of Botanical Medicine Refinement Engineering, Nanjing University of Chinese Medicine, Nanjing 210023, China*

*^c^ School of Chinese Materia Medica, Beijing University of Chinese Medicine, Beijing 102488, China*

*^d^ School of Chemical Engineering, The University of Queensland, St Lucia, QLD, 4072 Australia.*

Submitted to

*Advanced Science*

15 December 2025

Supplementary methods

1.1 Modeling

membrane models: PEI chains, composed of 14 monomers, were constructed. And ionization was performed on the seventh tertiary nitrogen atom of each the chain. Subsequently, the surface models with a thickness of 4 nm were generated by stacking the polymer chains using Packmol. Ultimately, the completed models were pretreated by water using a 5-cycle annealing scheme (300 K low temperature, 800 K high temperature).

Phytic acid was optimized using density functional theory (DFT) with the B3LYP-D3(BJ)/def2TZVP level. Atomic charges were fitted using Multiwfn to obtain RESP2 charge [1], and the topology files were generated with Sobtop. Water was represented using the three-point potential model. Details of ion models force field parameters are provided in Table 1.

1.2 Simulation Setting

MDS were performed and analyzed using GROMACS 2026.0, and visualizing the trajectories was conducted with VMD. The molecular topology was defined based on the GAFF. The cutoff distance for the non-bonded Lennard-Jones interaction was set at 1 nm. The Particle-Mesh Ewald (PME) method was employed to calculate the long-range electrostatic forces. The linear constraint solver (LINCS) algorithm was utilized to maintain covalent bonds involving H atoms at their equilibrium values. The NPT ensemble was employed in both the equilibrium and production runs in this study. The V-rescale method was employed to control the temperature at 298.15 K. The C-rescale method was utilized to keep the NPT ensemble at 1 bar.

1.3 The simulation of absorption behavior for water droplets on PEI membrane

A water droplet containing phytic acid was inserted 5 nm away from the membrane surface. The system was then solvated with n-hexane and charge neutralization was performed. Table 1 details the specific composition of the systems. All systems underwent 1 ns equilibrium runs to achieve stability (i.e., the overall energy and temperature exhibit small fluctuations) followed by 30 ns production runs for data collection. To maintain membrane structural stability, a positional restraint of 1000 kJ mol⁻¹ nm⁻² was applied along the z-axis during production runs, while atoms were allowed to move freely in the x–y directions.

Table S1 The information about ion models

| moleculetype | | | | | | | | | | | |
| --- | --- | --- | --- | --- | --- | --- | --- | --- | --- | --- | --- |
| molname | | | | | | nrexcl | | | | | |
| CL | | | | | | 1 | | | | | |
| atomtypes | | | | | | | | | | | |
| name | | at.num | | mass | | ptype | | sigma | | epsilon | |
| Cl | | 17 | | 35.45 | | A | | 4.40104e-1 | | 4.18400e-1 | |
| atoms | | | | | | | | | | | |
| id | at type | | res nr | res name | at name | | cg nr | | charge | | mass |
| 1 | Cl | | 1 | CL | CL | | 1 | | -1.00000 | | 35.45 |

Table S2 Arrangement of simulation of absorption behavior for water droplets on PEI membrane

| System | Number |
| --- | --- |
| PA | 10 |
| Water molecules | 3765 |
| PEI | 250 |
| Hexane | 20394 |
| Chloride ions | 180 |

Supplementary results


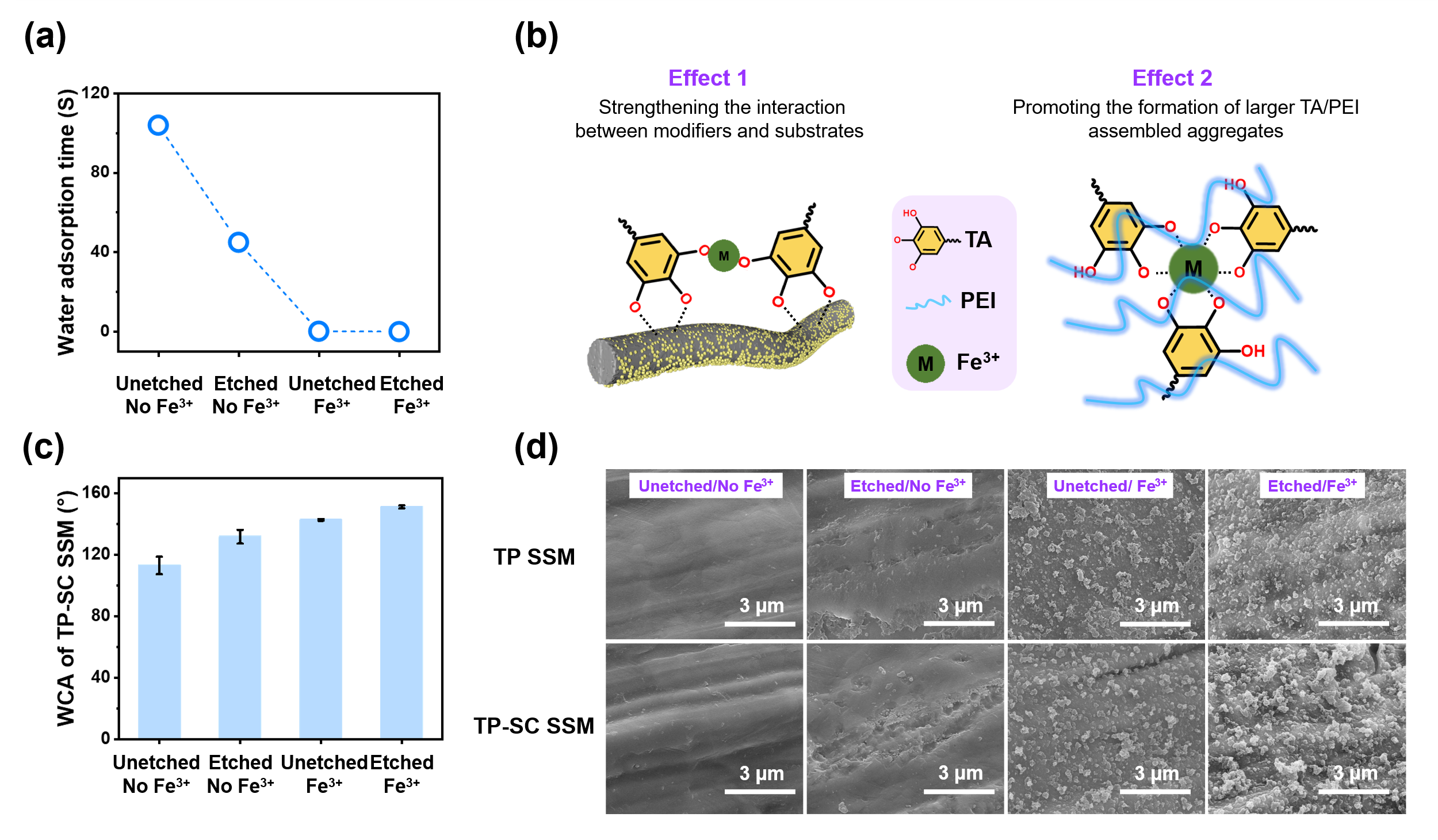


Figure S1. The effect of etching and Fe^3+^ on the surface morphology of TP-SC SSM. All SSM was thoroughly washed after etching step to remove residual Fe^3+^.


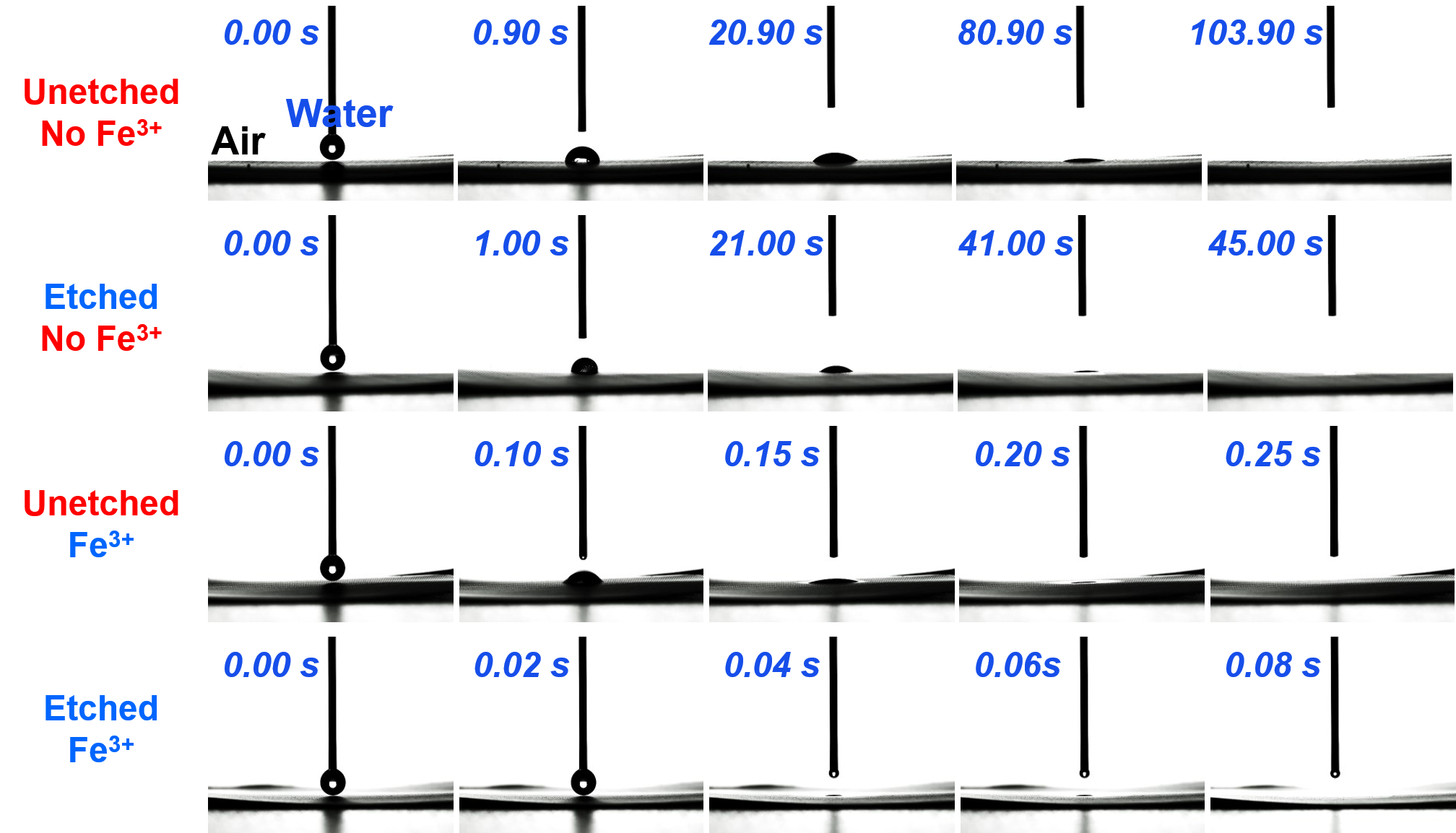


Figure S2. The water adsorption time of TP SSM based on Unetched, Etched/No Fe^3+^ addition, Unetched/Fe^3+^ addition, and Etched/Fe^3+^ addition method, respectively. All SSM was thoroughly washed after etching step to remove residual Fe^3+^.


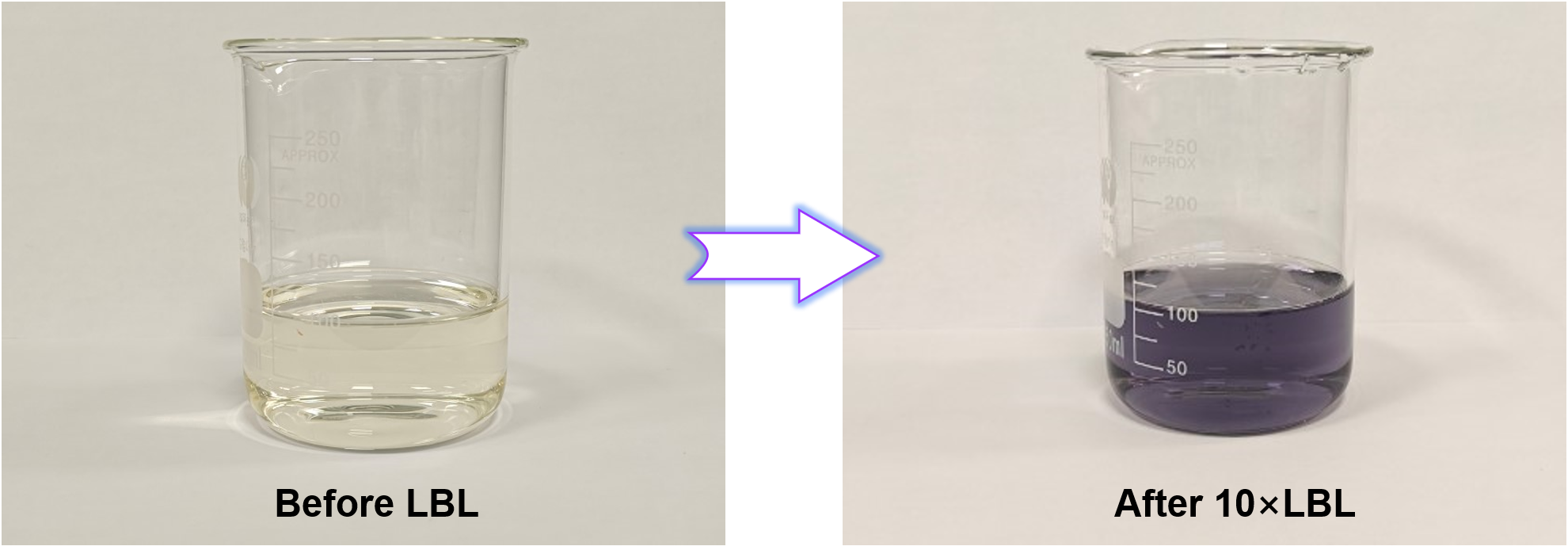


Figure S3. The color change of TA solution in LBL process of etched and mildly cleaned SSM.

To investigate the effects of etching and residual Fe^3+^ in LBL process, LBL was carried out in Unetched, Etched/No Fe^3+^ addition, Unetched/Fe^3+^ addition, and Etched/Fe^3+^ addition method. Initial attempts confirmed the crucial role of etching, as deposition onto unetched SSM yielded almost no TP aggregates and poor hydrophilicity and hydrophobicity for TP and TP-SC SSM, respectively (Figure S1 and Figure S2). However, etching alone proved insufficient; substrates etched but vigorously rinsed (removing possible residual Fe^3+^) lacked the clear hierarchical structure seen in optimized samples (similar to original SSM) and exhibited unsatisfactory wettability.

The key discovery was the essential role of trace Fe³⁺ ions, retained via mild washing after etching (Figure S3). After the introduction of Fe^3+^ in TA solution, despite the absence of etching step, more and larger LBL aggregates emerged and wettability changed dramatically. The combination of etching and Fe^3+^ addition synergistically promotes TP LBL deposition, enhancing modifier-substrate interaction while fostering the formation of larger TA/PEI aggregates (Figure S1b). Consequently, the optimized approach successfully established the essential hierarchical architecture. This structure enabled the resulting TP platform to exhibit super-hydrophilicity (water absorption < 0.02 s) and the TP-SC platform to achieve robust super-hydrophobicity (> 150°), forming the ideal basis for subsequent constructing JMs.

Table S3 The concentration of Fe^3+^ in TA solution of etched and mildly washed SSM.

|  | Sample 1 | Sample 2 | Sample 3 |
| --- | --- | --- | --- |
| Fe^3+^ (mM) | 0.057 mM | 0.059 mM | 0.046 mM |


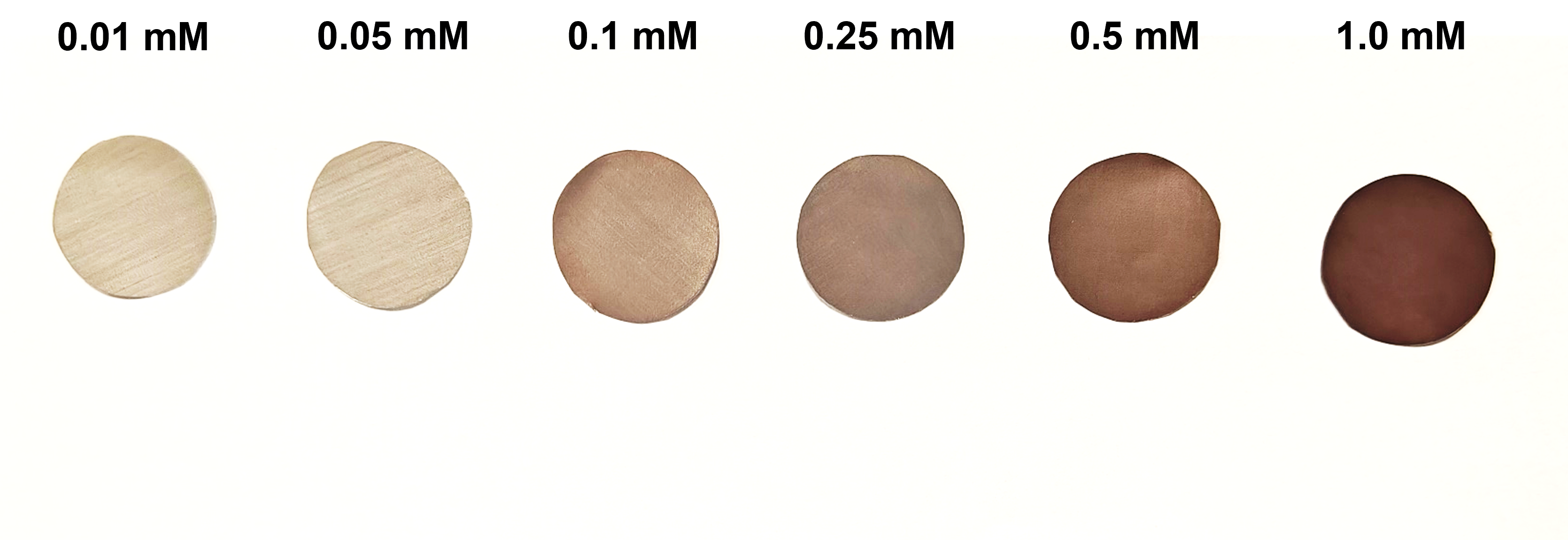


Figure S4. The influence of Fe^3+^ in TA solution on the appearance of TP SSM.


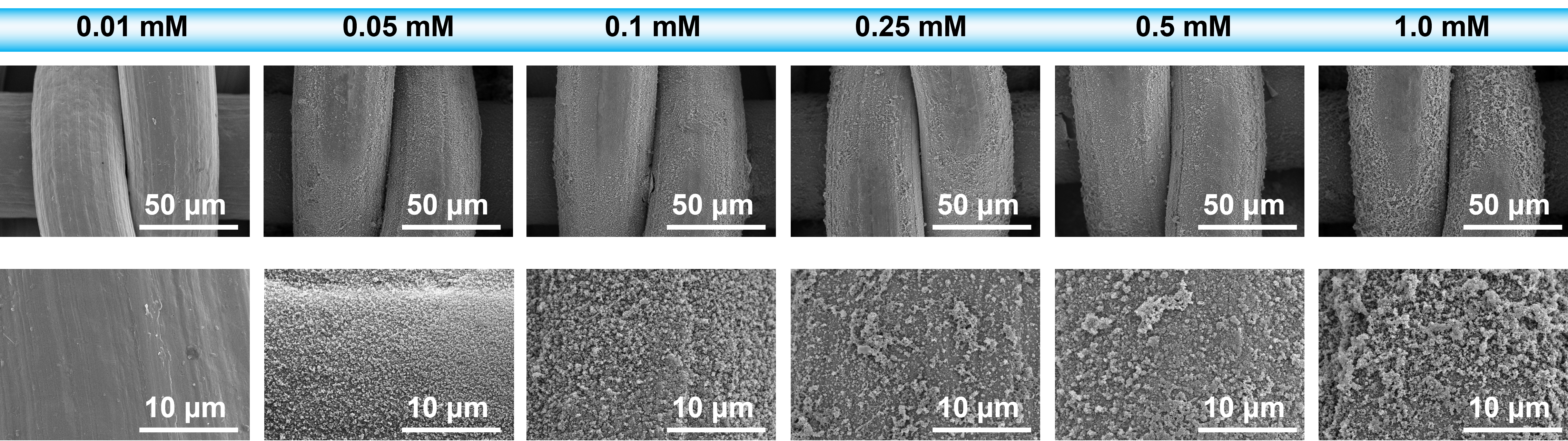


Figure S5. The influence of Fe^3+^ in TA solution on the morphology of micro-nano structures in TP SSM.


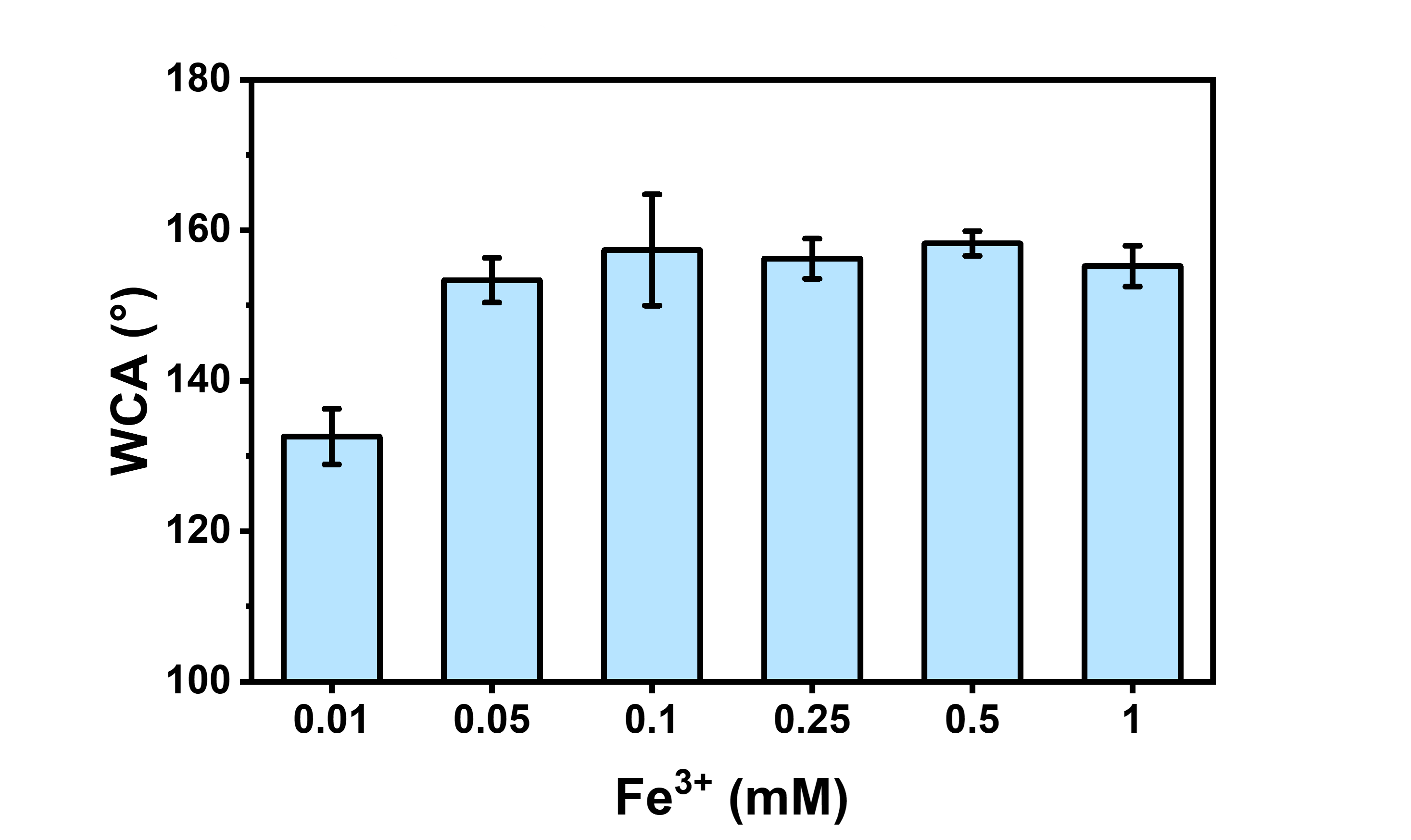


Figure S6. The influence of Fe^3+^ in TA solution on the hydrophobicity of TP-SC SSM.

Considering the beneficial effect of Fe^3+^ on micro-nano structure formation, we measured the concentration of Fe^3+^ by ICP-MS (Agilent 7850). Specifically, the etched SSM was mildly washed by water flow and subsequently soaked in deionized water for 5 min accompanied by constant shaking, ensuring Fe^3+^ are thoroughly dispersed. Moreover, we conducted a detailed investigation into the influence of Fe^3+^ concentration in the TA on membrane microscopic morphology and wettability. As shown in Figure S4, the membrane color gradually changed to reddish-brown with increasing Fe^3+^ concentration. Figure S5 demonstrates that adding Fe^3+^ at concentrations ranging from 0.05 mM to 1.0 mM effectively promotes the formation of micro-nano structures on the SSMs. Conversely, excessively low Fe^3+^ concentrations, such as 0.01 mM, fail to provide adequate promotion of LBL, resulting in rare micro-nano structure formation and poor hydrophobicity (Figure S6). Therefore, when preparing TP-based substrate films, it is essential to ensure the presence of an appropriate amount of Fe^3+^ in the TA.


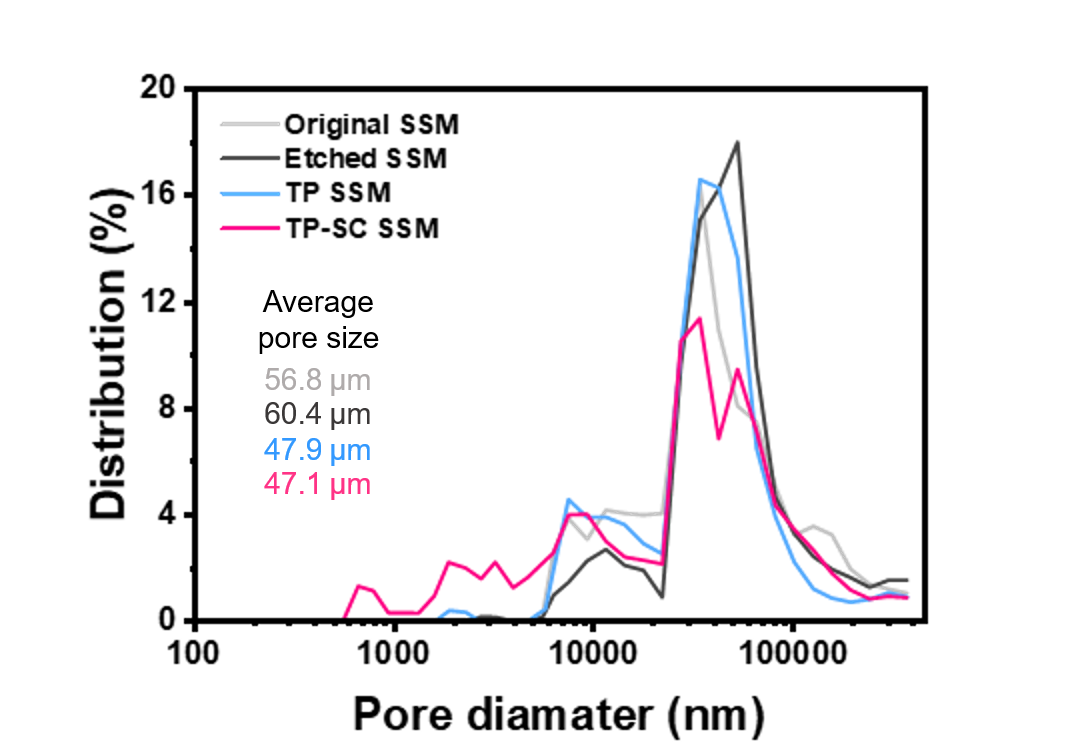


Figure S7. Statistical pore size distribution of original, etched, TP, and TP-SC SSM.


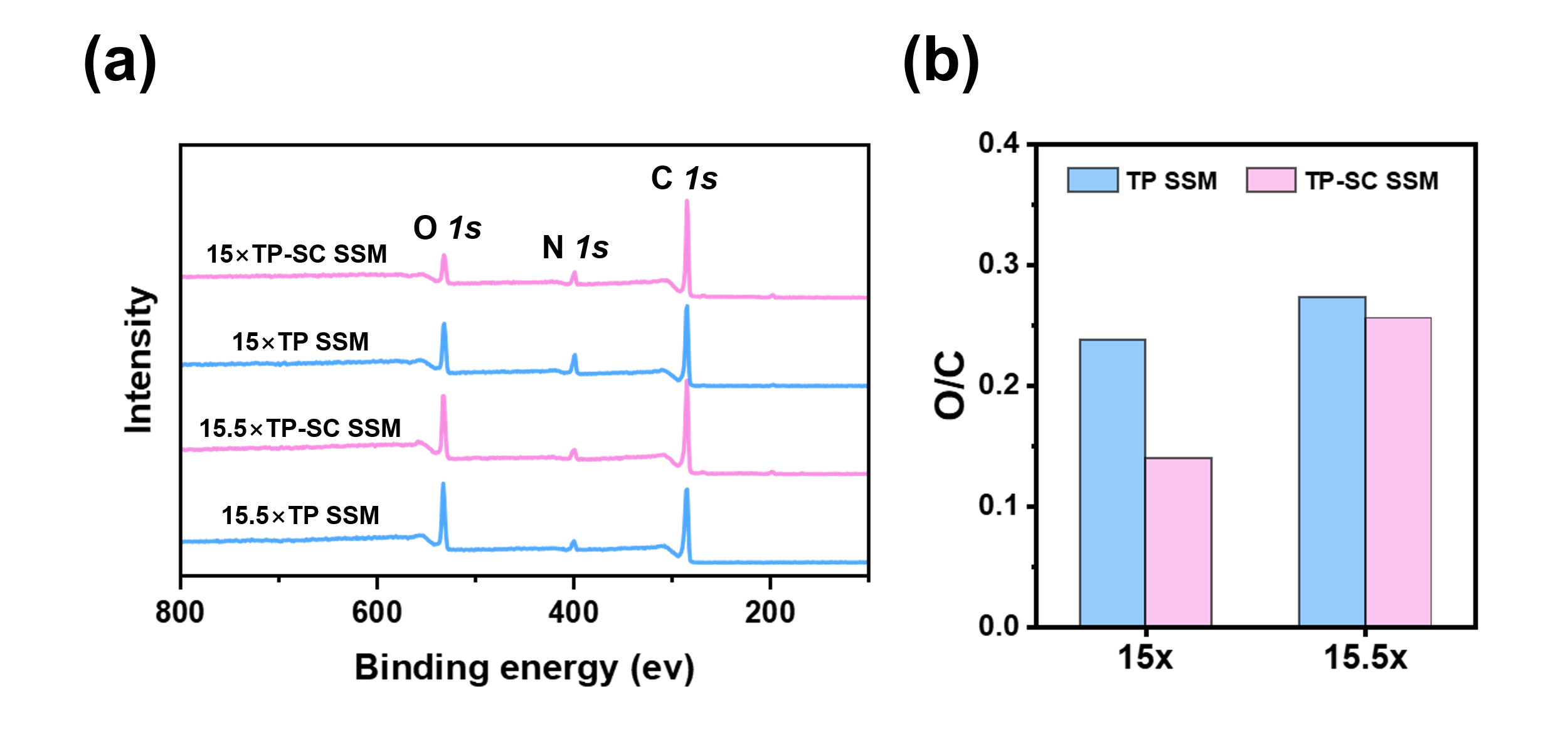


Figure S8. (a) The XPS wide-scan spectra and (b) O/C of 15×TP, 15×TP-SC, 15.5×TP, and 15.5×TP-SC SSM, respectively.


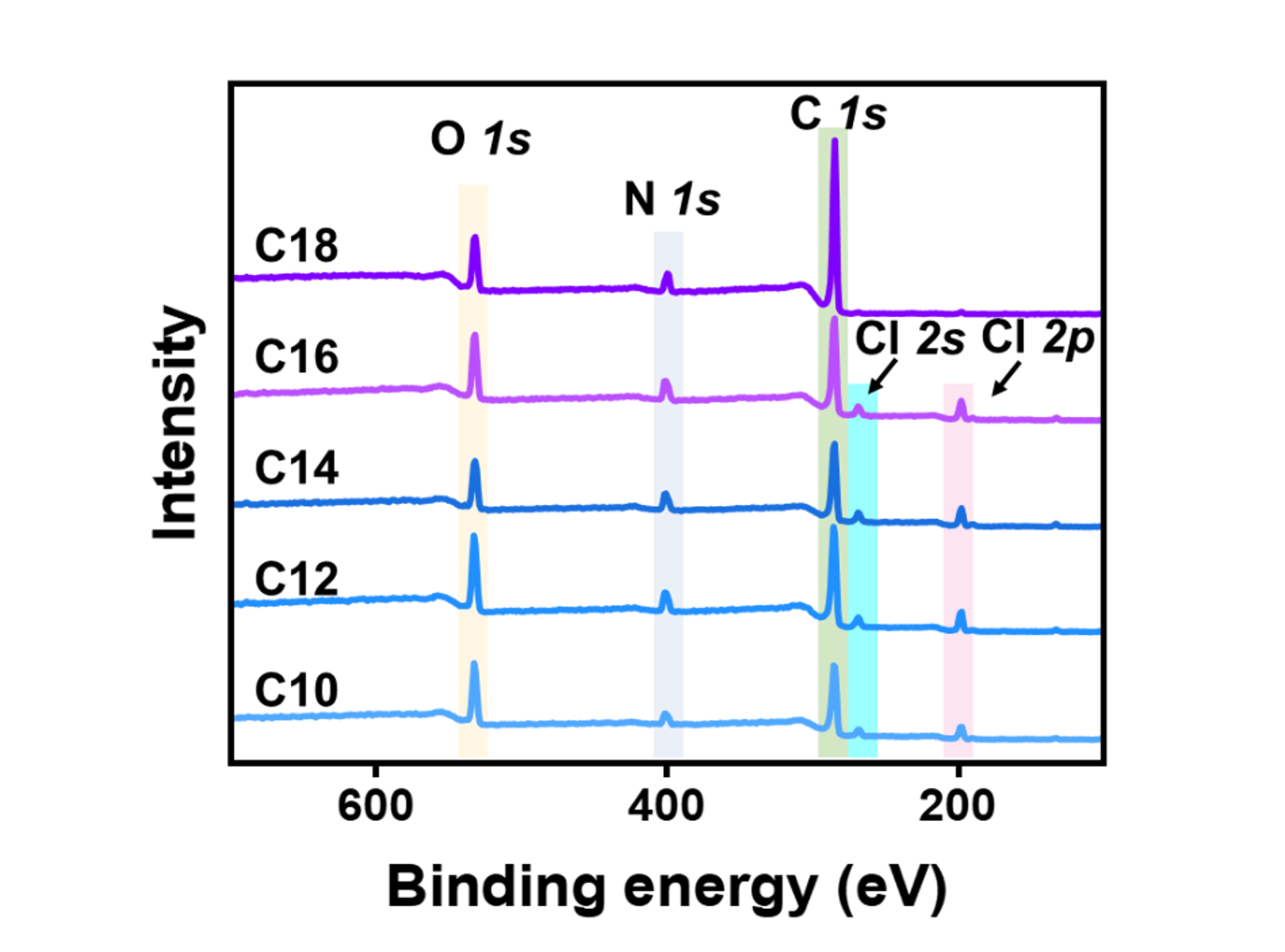


Figure S9. The influence of alkyl acyl chlorides with different lengths of hydrophobic chains on hydrophobic performance and X-ray photoelectron spectroscopy (XPS) results.


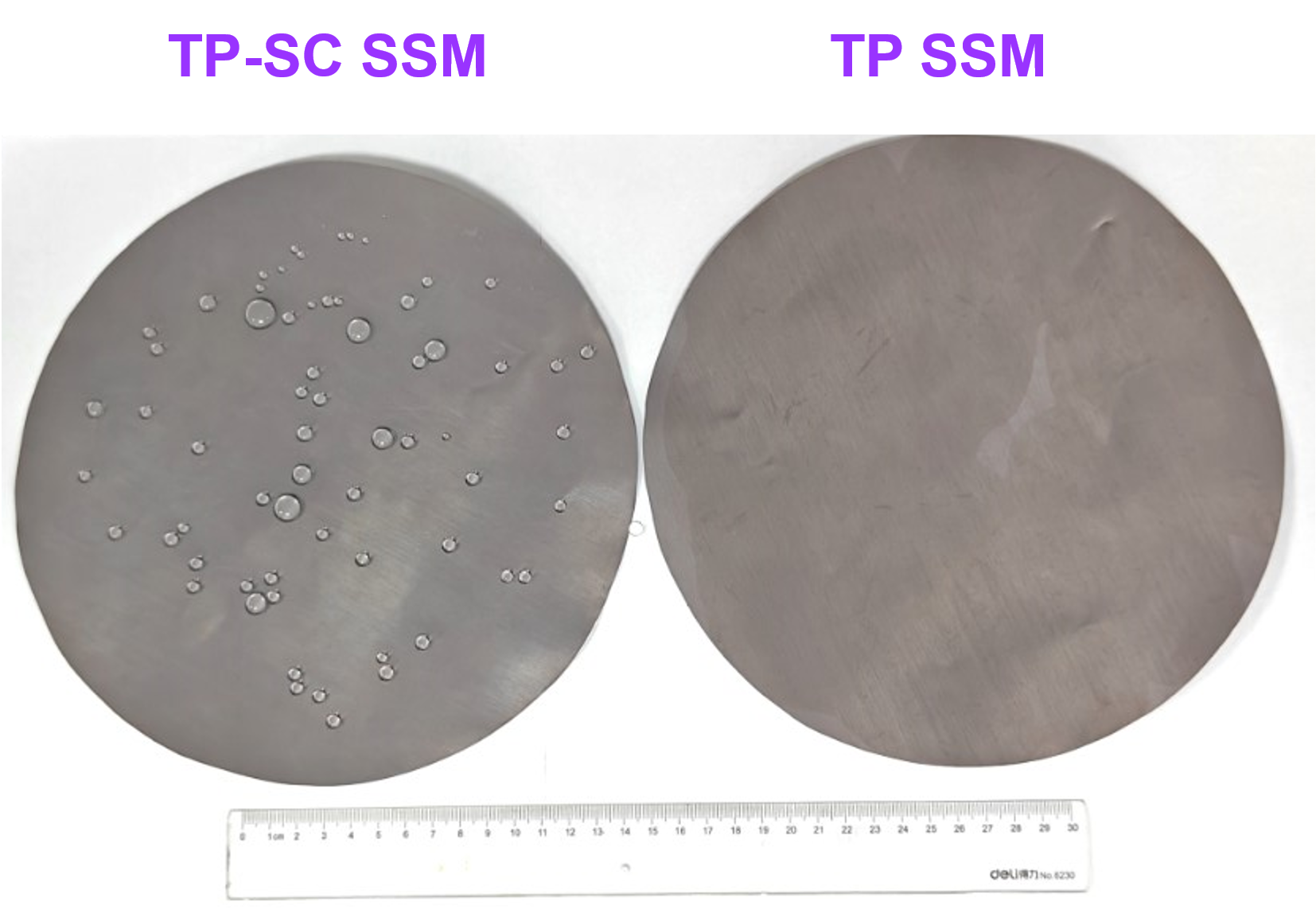


Figure S10. Photograph of TP and TP-SC SSM with diameter of 23 cm.


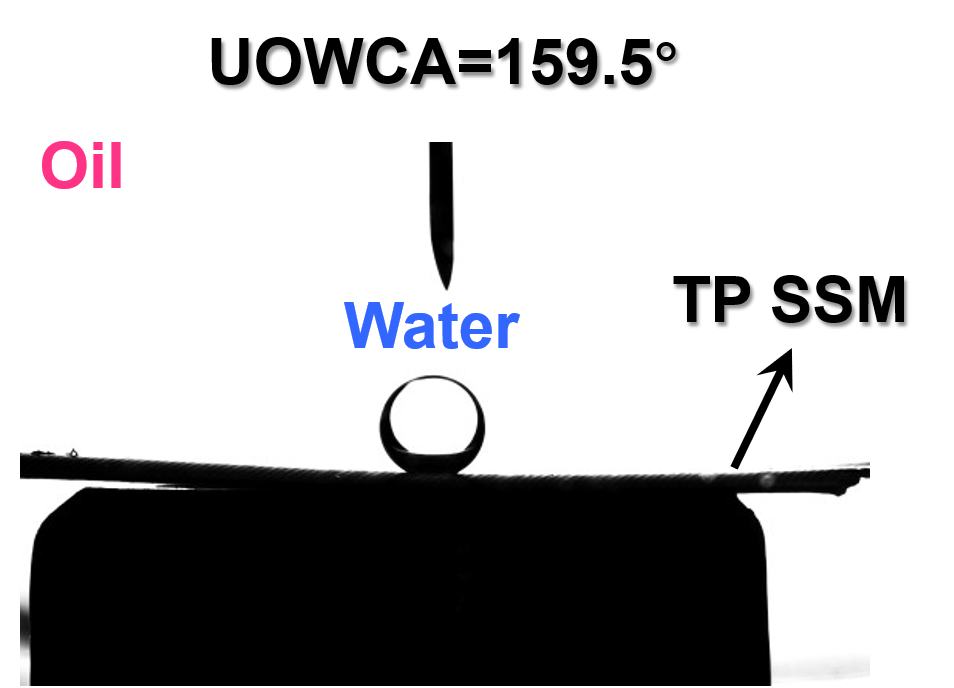


Figure S11. The state of TP SSM in water-hexane interface. Hexane is stained with Oil Red O, while water is stained with Methylene Blue.


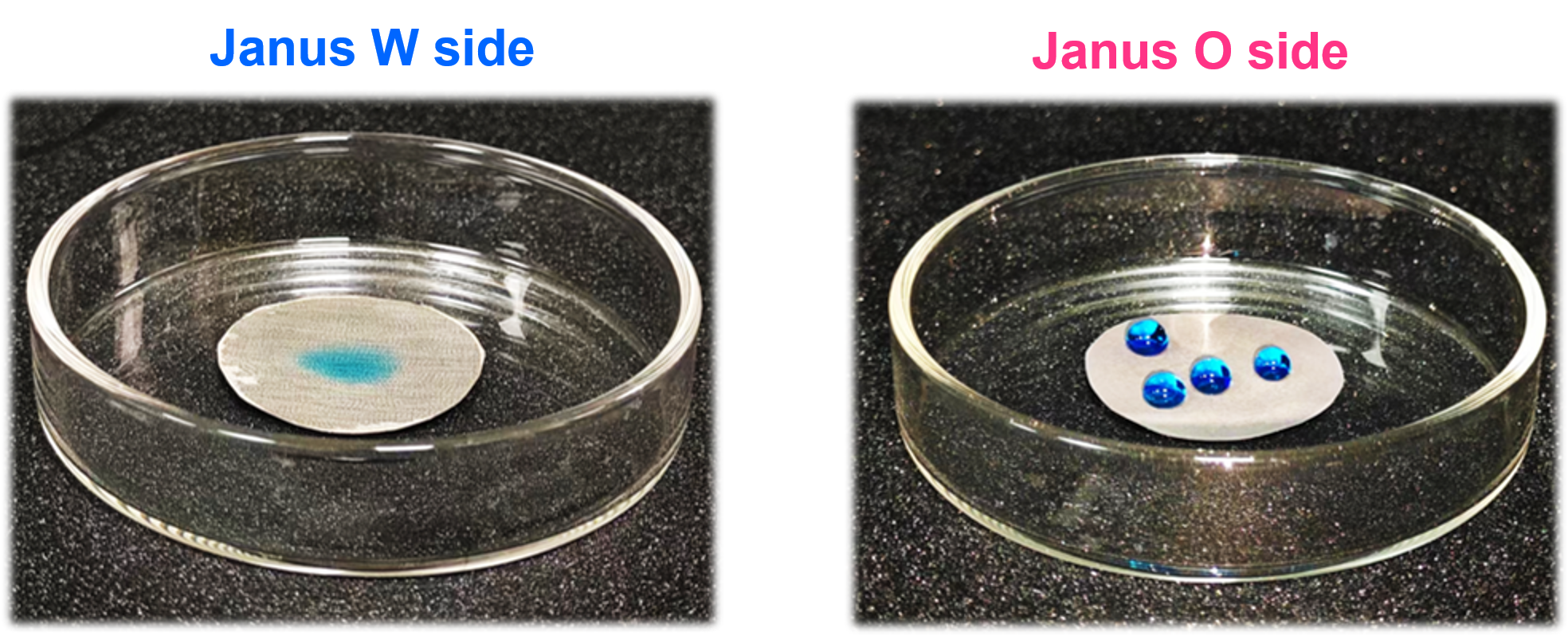


Figure S12. The wettability of JMs.


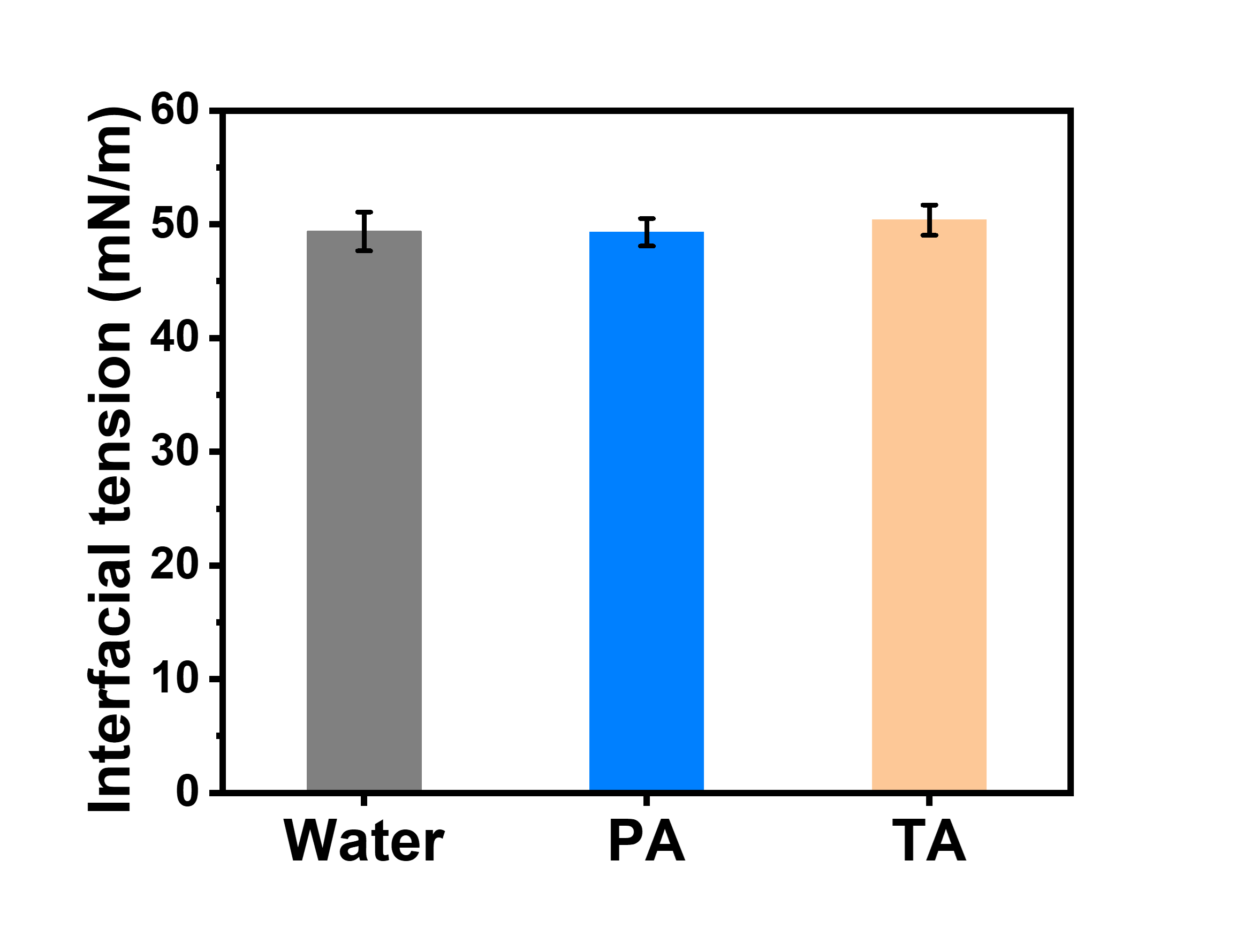


Figure S13. The effect of solutes on the water-hexane interfacial tensions. Pendant droplet technique [2] was employed to analyze droplets of water, PA solution, and TA solution. suspended in hexane.


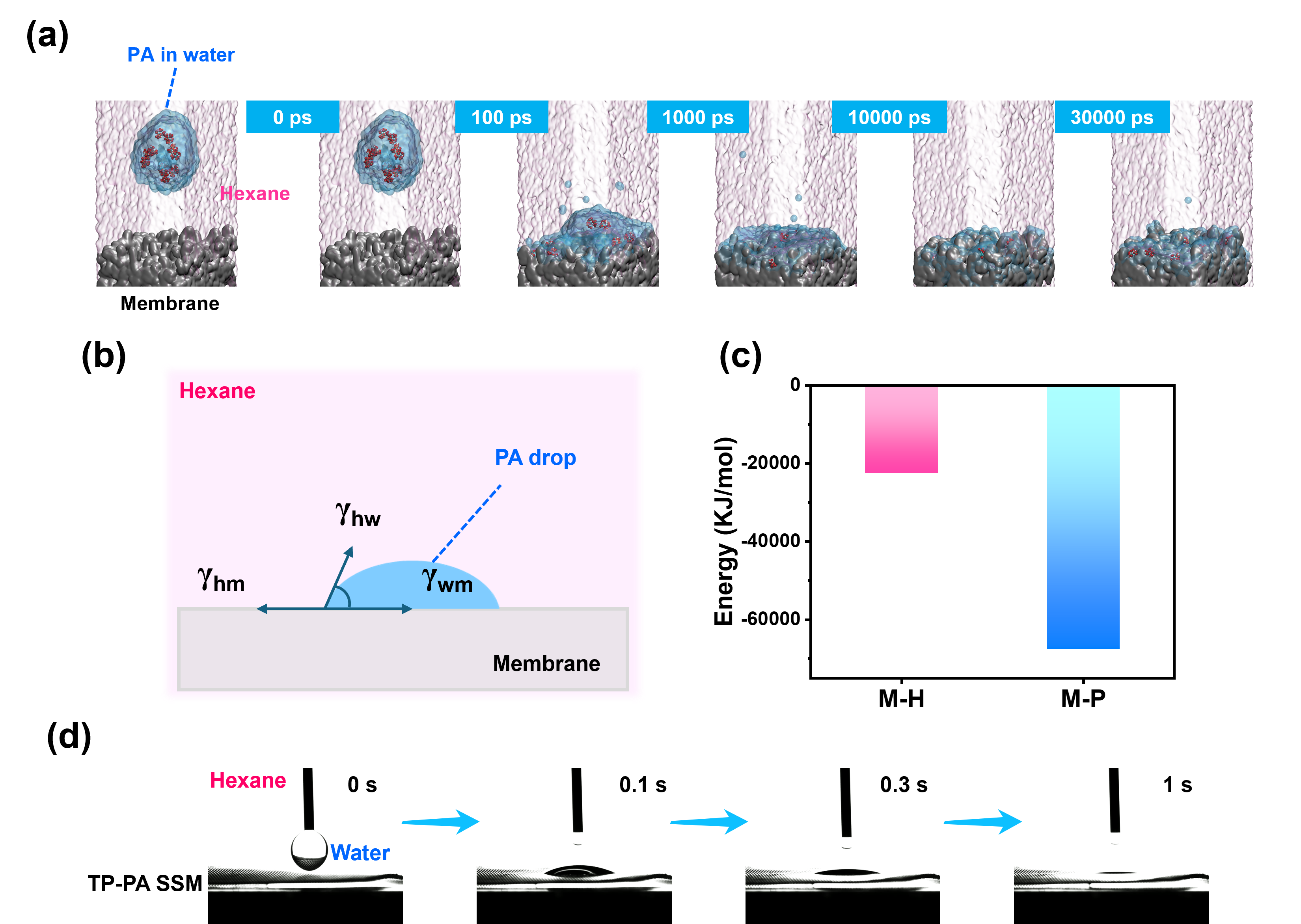


Figure S14. (a) Snapshot of MD simulation for PA droplet wetting process on TP membrane in n-hexane. (b) Schematic of PA drop on TP membrane in hexane. (c) The interaction energy between membrane-hexane (M-H) and membrane-PA drop (water and PA) (M-P), respectively. (d) Wetting behavior of TP membrane adsorbed with PA (TP-PA) in n-hexane.


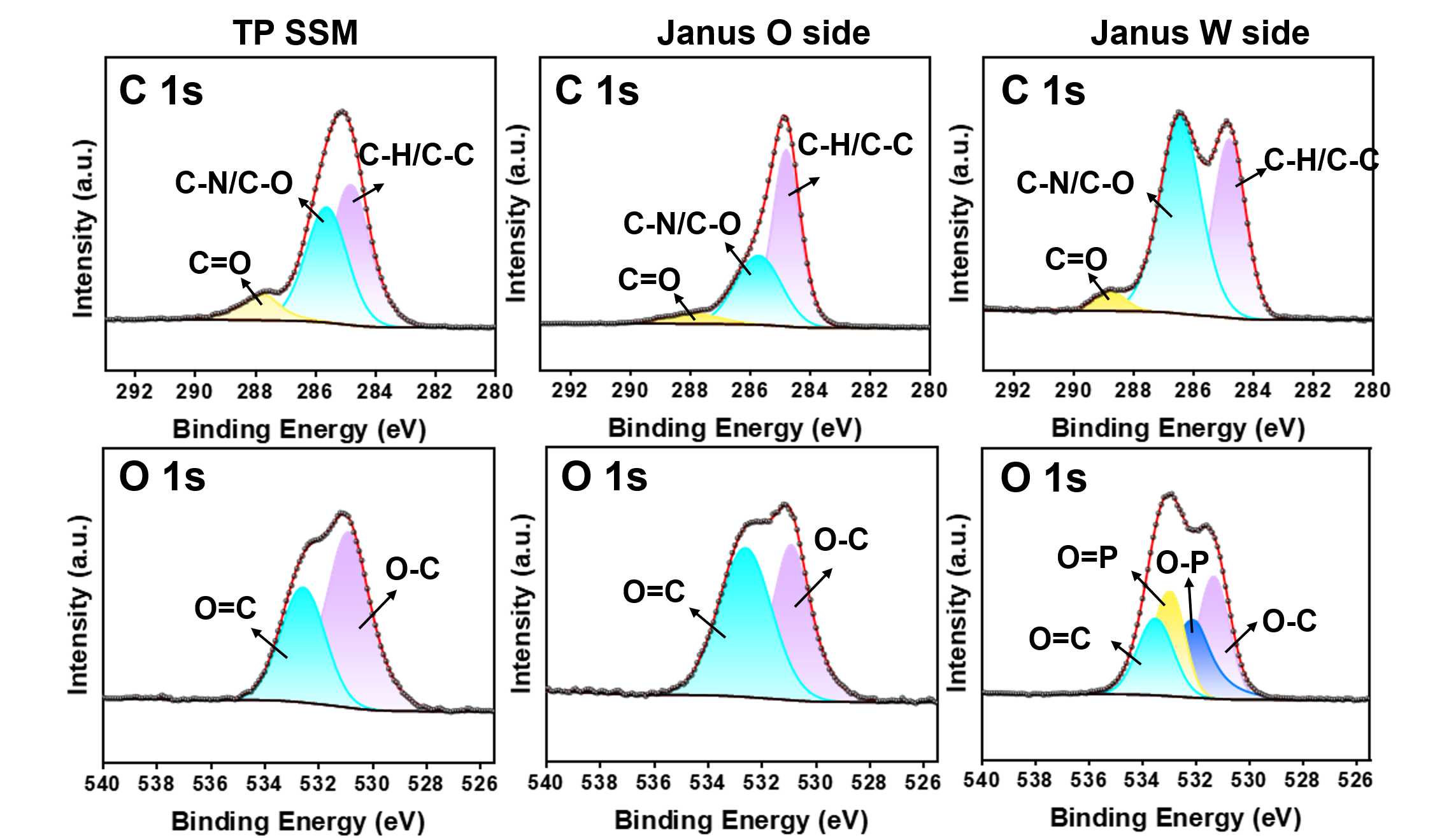


Figure S15. XPS spectra of TP SSM, Janus O side of JMs, and W side of JMs.

The content of C-O separated from C 1s spectra in Janus W side obviously higher than TP SSM. Moreover, in addition to O-C and O=C, O-P and O=P were also separated from the narrow-scan spectra of O 1s in Janus W side. These results demonstrated that the successful modification of PA in Janus W side. In Janus O side. The content of both C-C separated from C 1s and O=C from O 1s increased compared to TP SSM, owing to the long hydrophobic chain of SC and formation of amide bond.


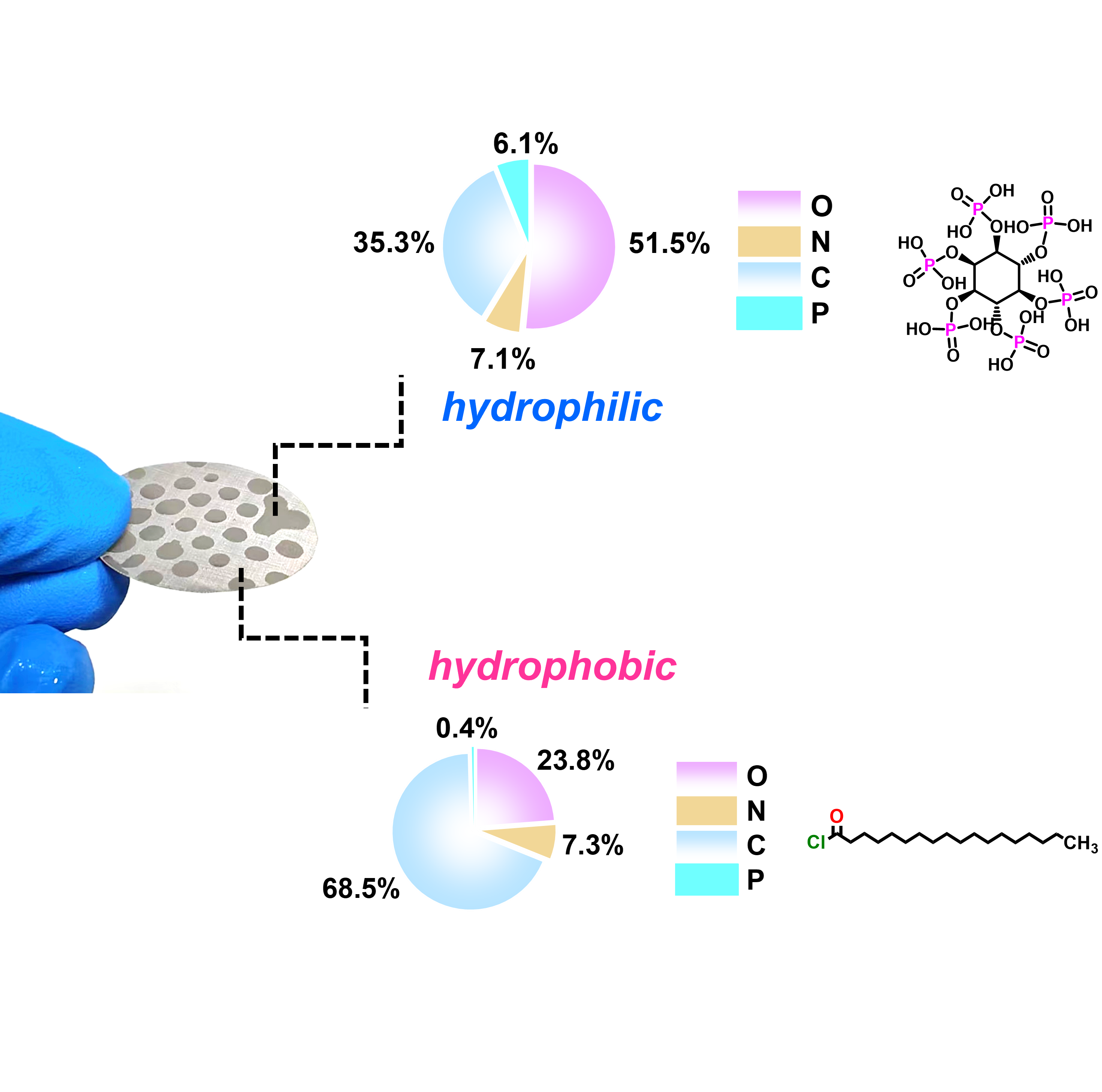


Figure S16. The elemental composition of hydrophilic and hydrophobic region in the heterogeneous wetting membrane fabricated by mask strategy.


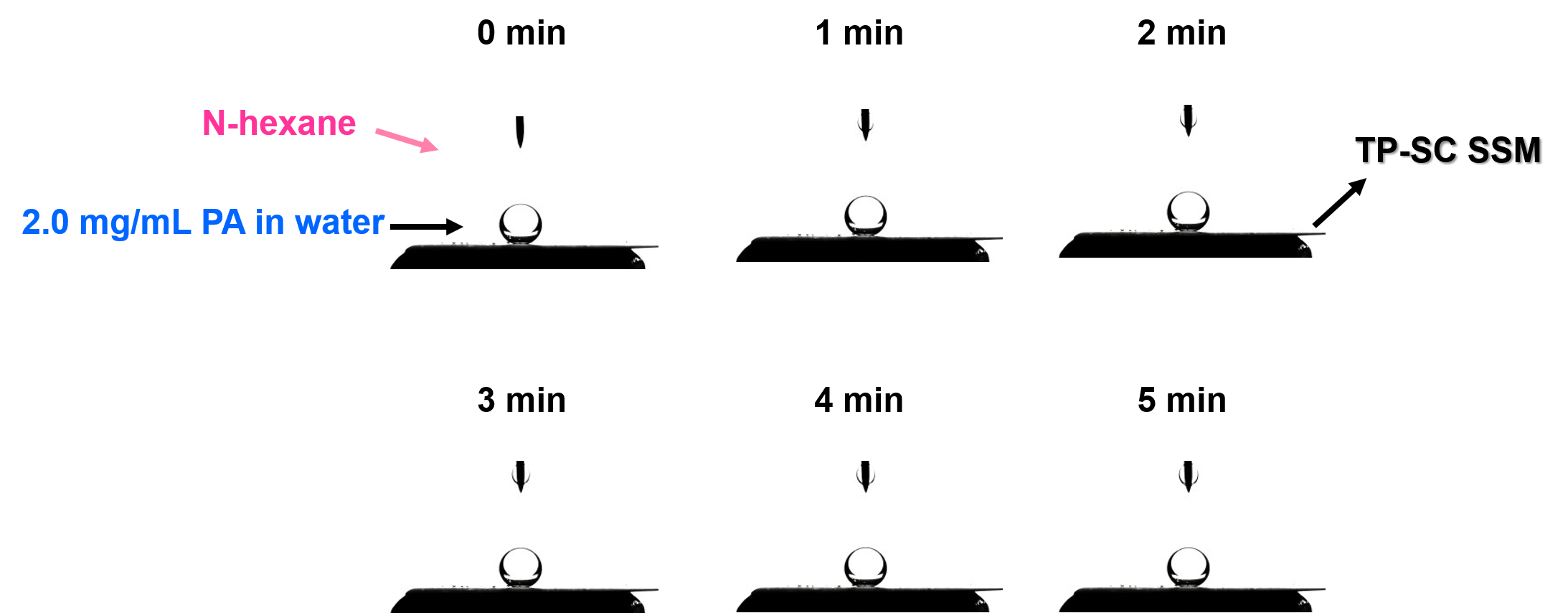


Figure S17. The UOWCA of TP-SC SSM. The water drop contains 2 mg/mL PA.


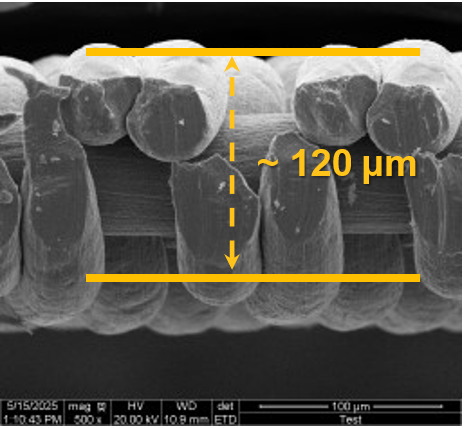


Figure S18. SEM image of the cross-section of JMs


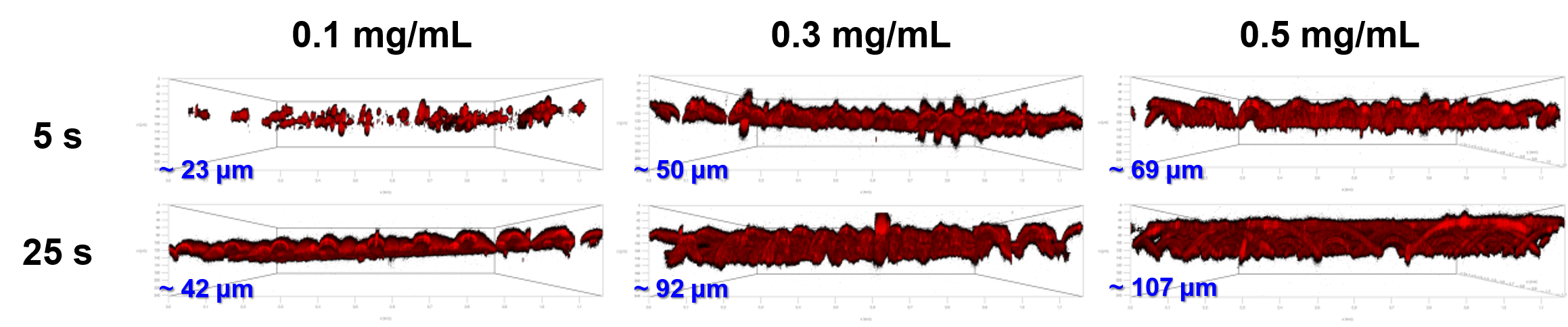


Figure S19. Images of the cross-sections of JMs with different thickness of hydrophilic layer.


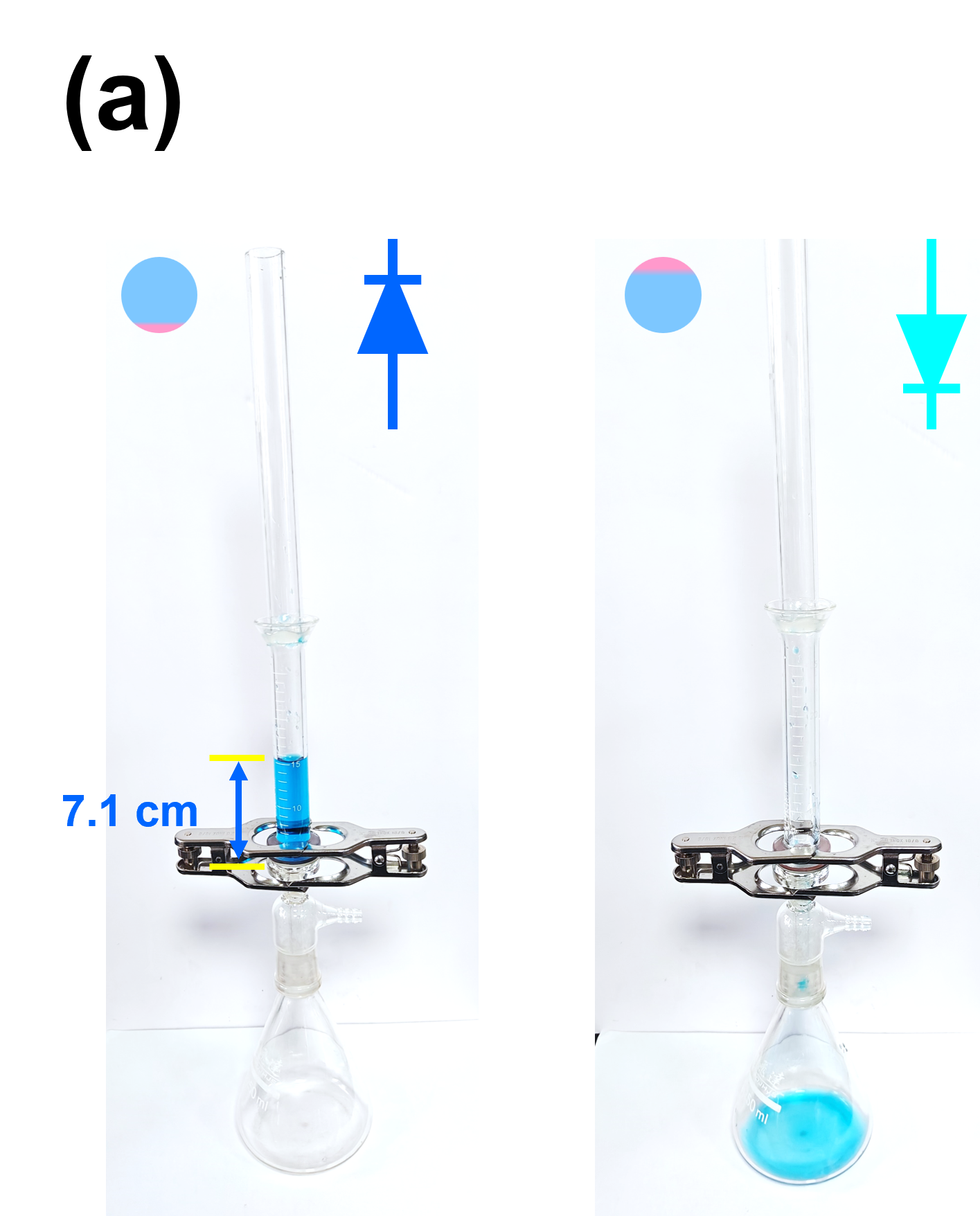


Figure S20. The unidirectional liquid transport performance of JMs prepared by 0.3 mg/mL PA and 25 s of SC addition time.


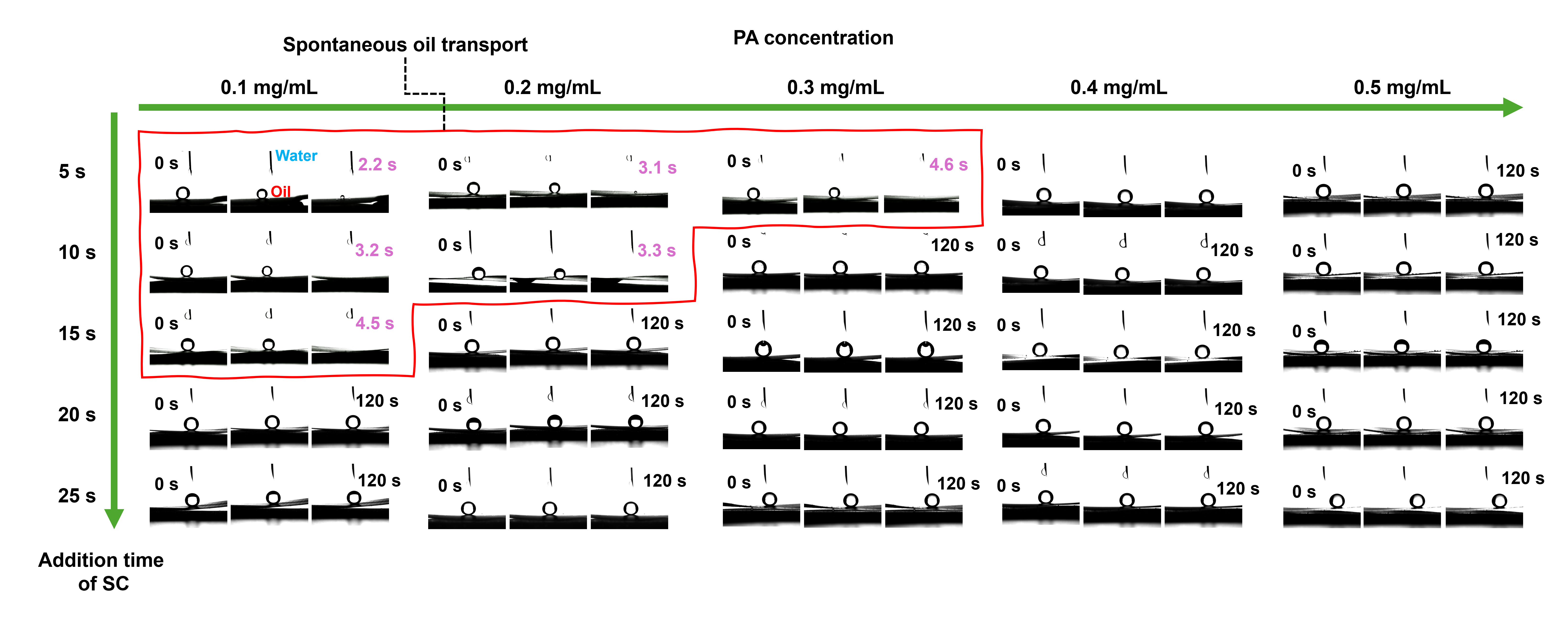


Figure S21. The oil transport behavior under water of JMs prepared by different PA concentration and SC addition time.


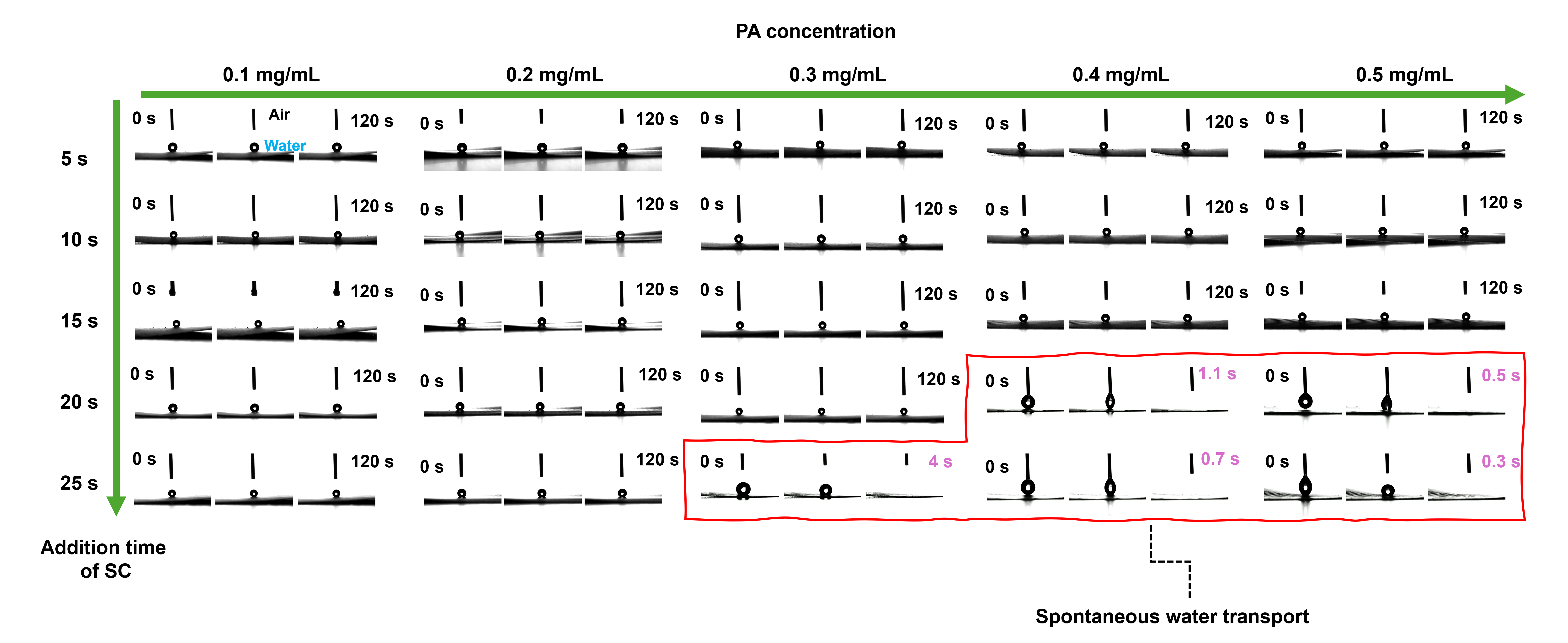


Figure S22. The water transport behavior of JMs prepared by different PA concentration and SC addition time.


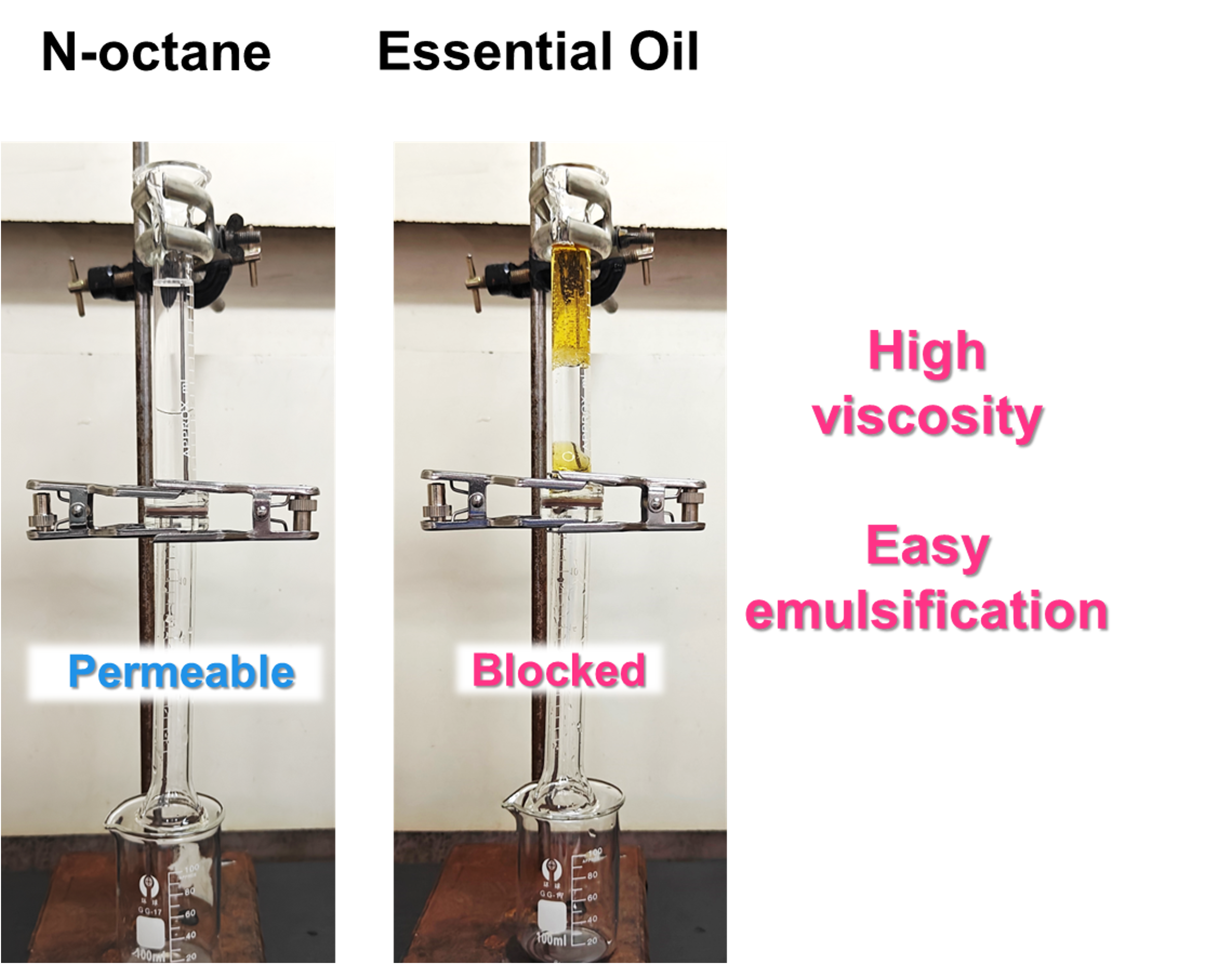


Figure S23. The state of mixture of n-octane/water and essential oil/water in a column-shape device.


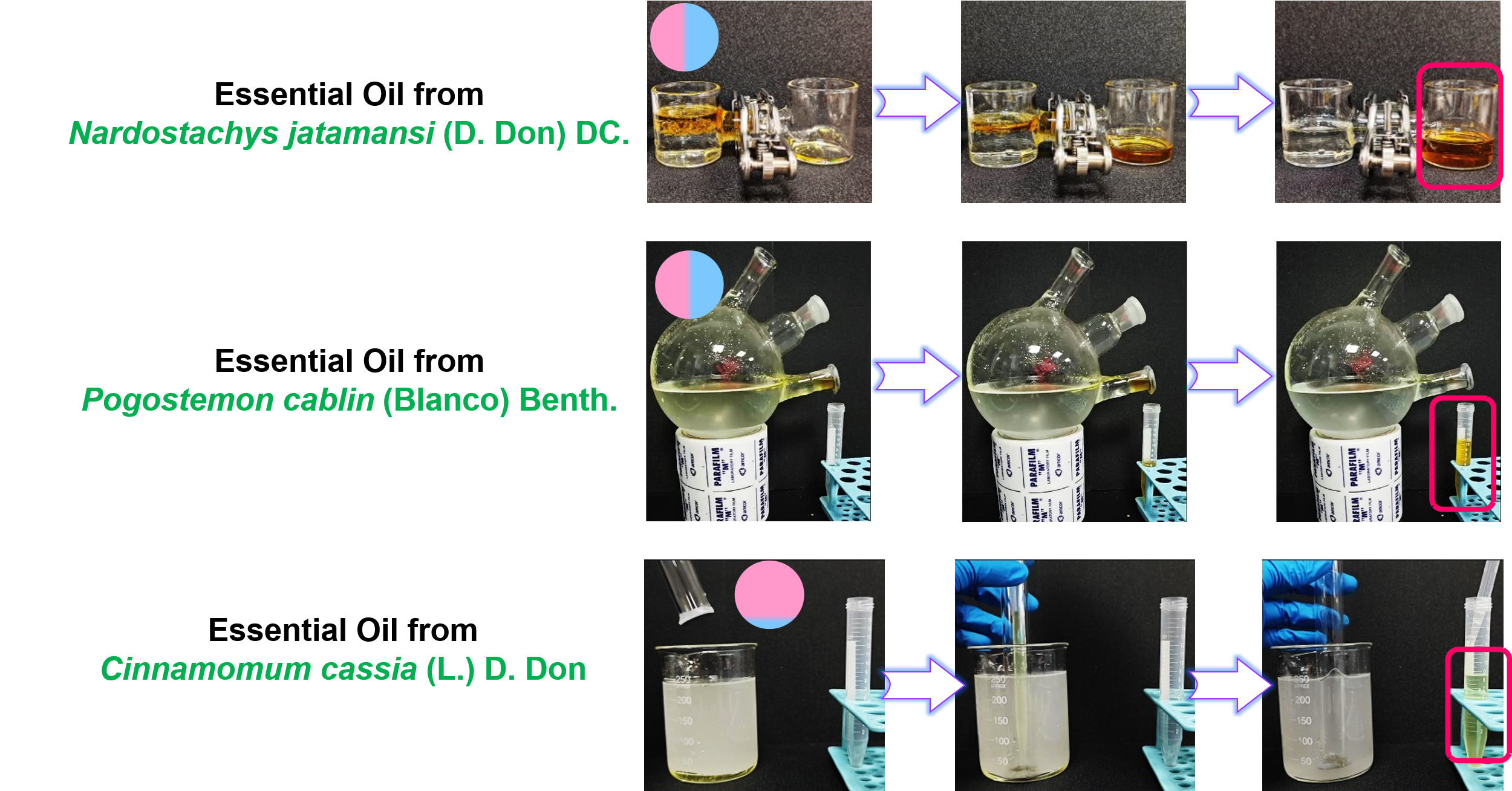


Figure S24. The separation of water and essential oil from *Nardostachys jatamansi* (D. Don) DC., *Pogostemon cablin* (Blanco) Benth., and *Cinnamomum cassia* (L.) D. Don by JMs.

When recovering essential oil from *Nardostachys jatamansi* (D. Don) DC. using an H-type apparatus, the oil readily permeates from Janus O side to W side while water is retained (Figure S24). Similarly, a JM fixed in three-neck flask successfully achieved the recovery of the oil phase floating atop the distillate of *Pogostemon cablin* (Blanco) Benth. Some plant-derived essential oils have densities greater than water and thus remain below the aqueous phase in the distillate. Therefore, JMs with thin hydrophilic/ thick hydrophobic layer can be employed for oil from *Cinnamomum cassia* (L.) D. Don recovery. The oil phase beneath the water surface was gradually collected into glass tubes.


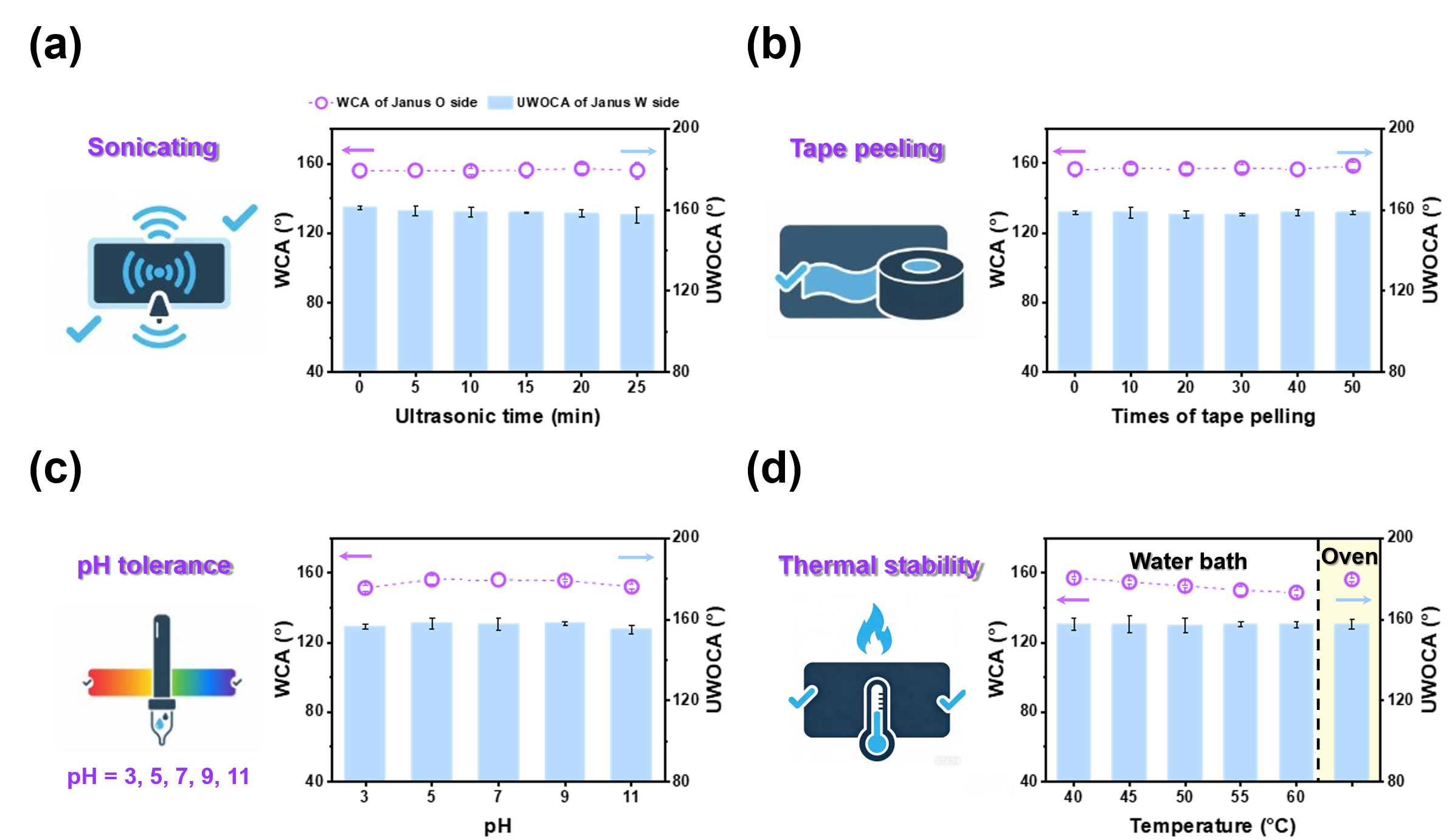


Figure S25. Mechanical, chemical, and thermal stability of JMs. The WCA of Janus O side and UWOCA of Janus W side after the damaging test of (a) sonicating for 0~25 min, (b) tape peeling for 0~50 times, (c) chemical solution treatment for 2 h, and (d) thermal incubation in water bath and oven for 2 h.


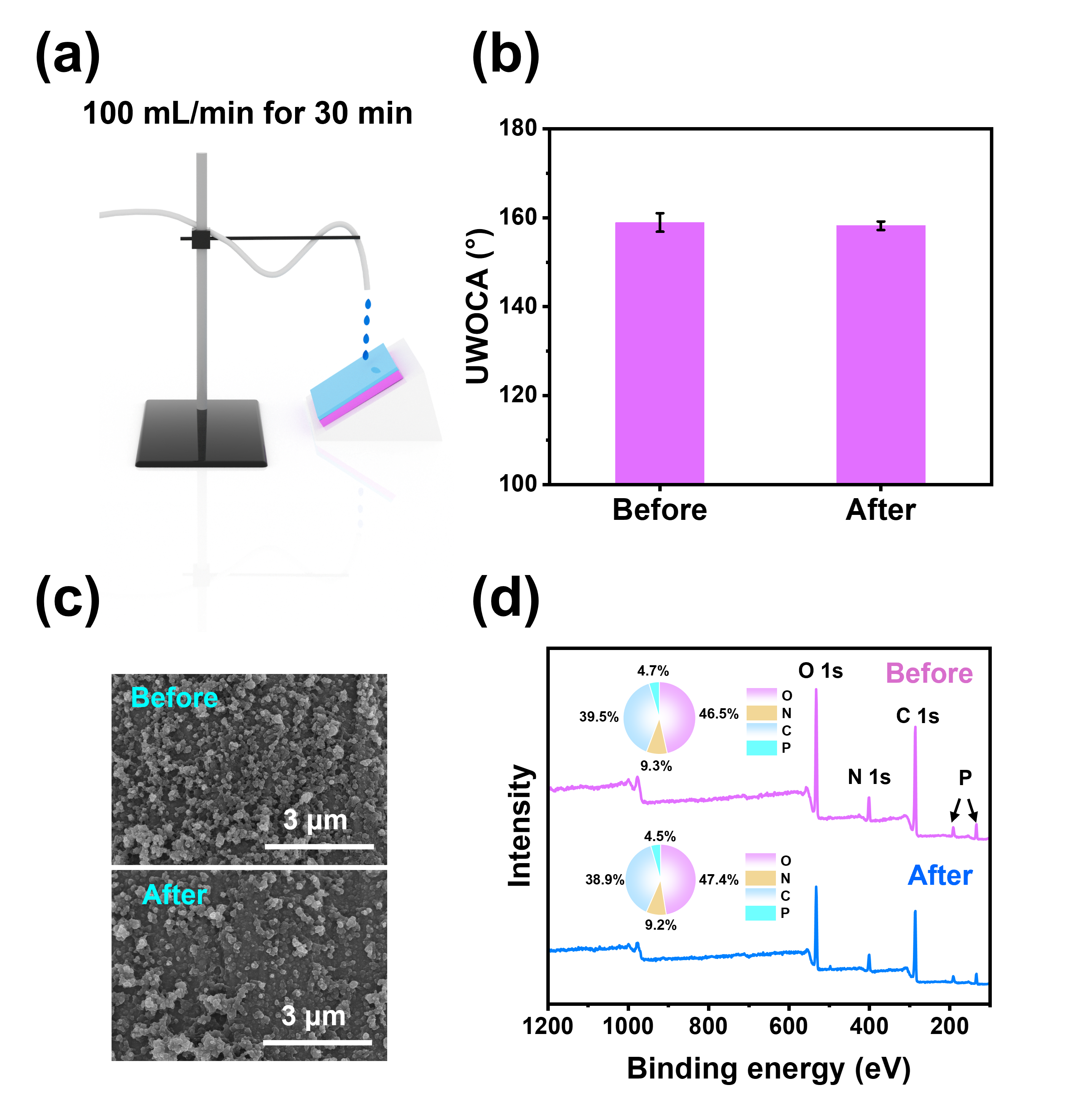


Figure S26. Flushing resistance of JMs. (a) Schematic Diagram of rigorous continuous flushing. The impact of water flushing on (b) UWOCA of Janus W side, (c) morphology of micro-nano structures and (d) PA adsorption.


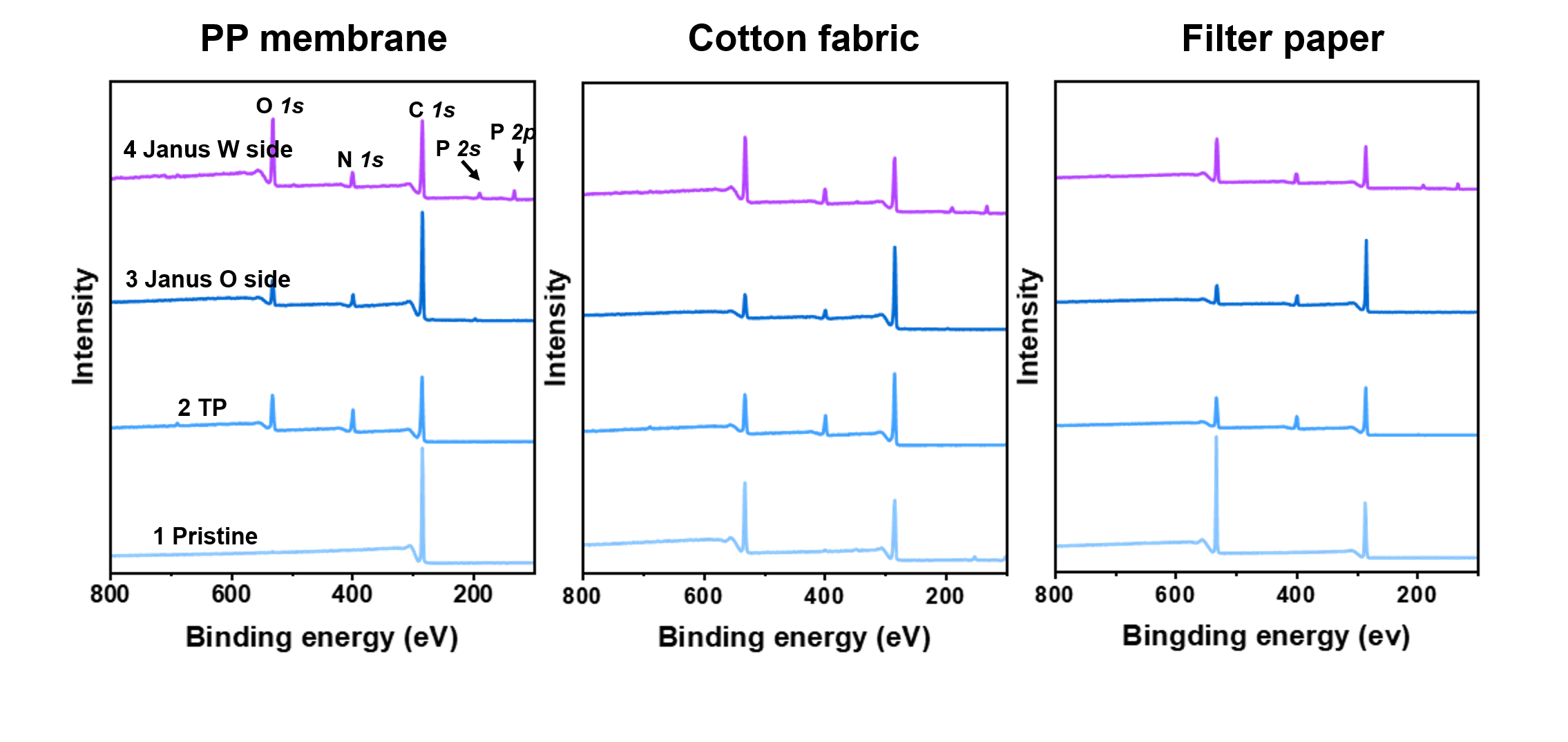


Figure S27. The XPS wide-scan spectra of pristine, TP, Janus O side, and Janus W side of PP membrane, cotton fabric, and filter paper, respectively.


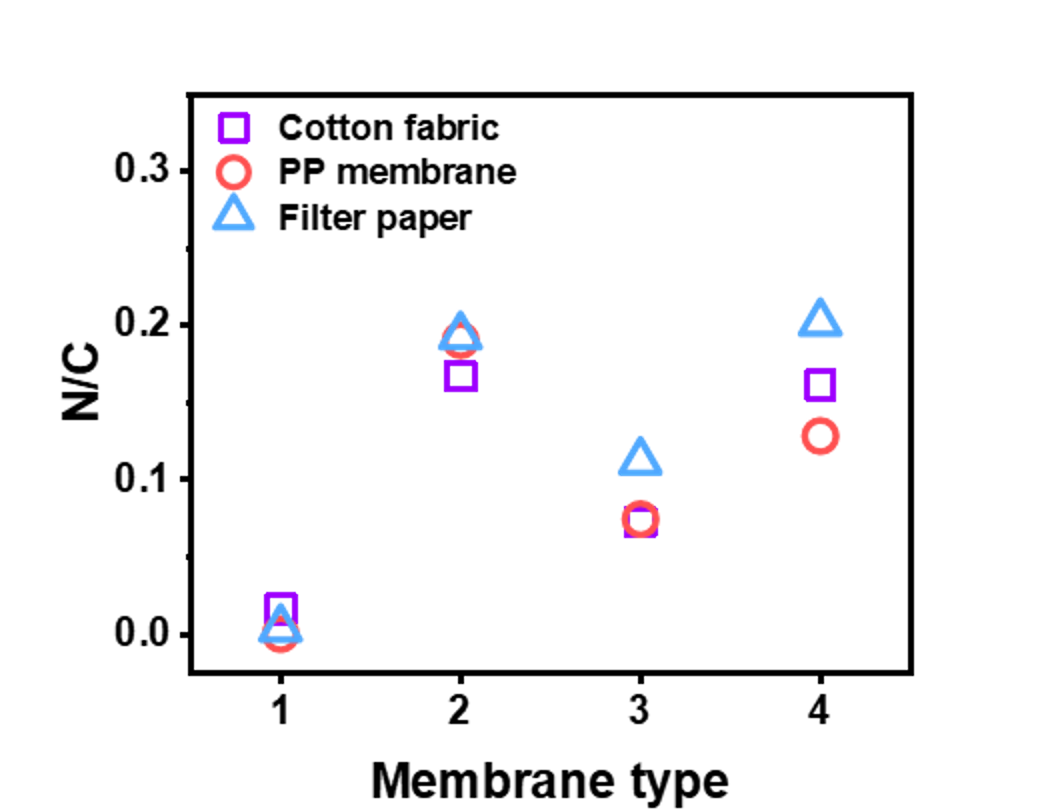


Figure S28. The N/C of pristine (1), TP (2), Janus O side (3), and Janus W side (4) of PP membrane, cotton fabric, and filter paper, respectively.


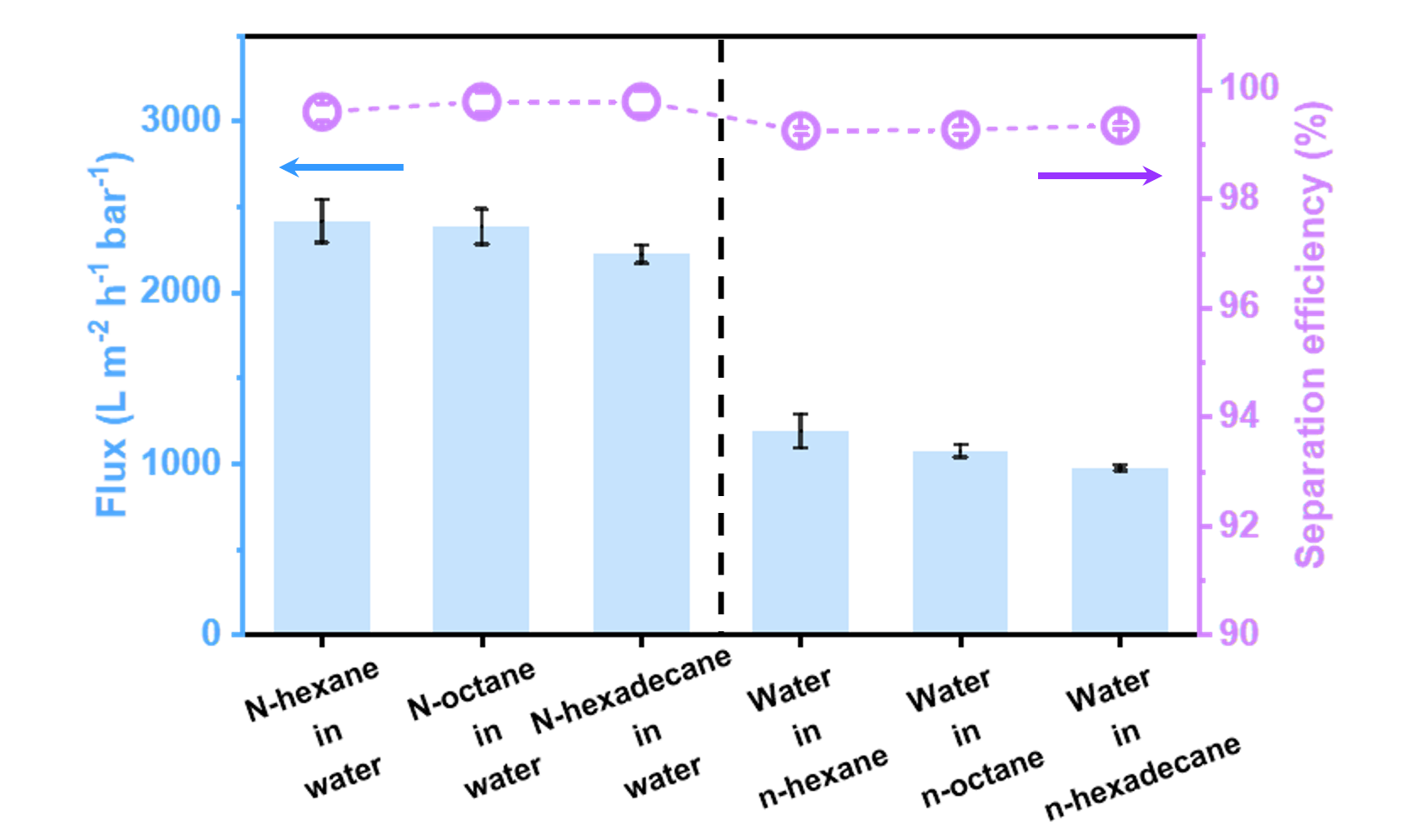


Figure S29. The excellent separation efficiency of JMs for W/O and O/W emulsion.


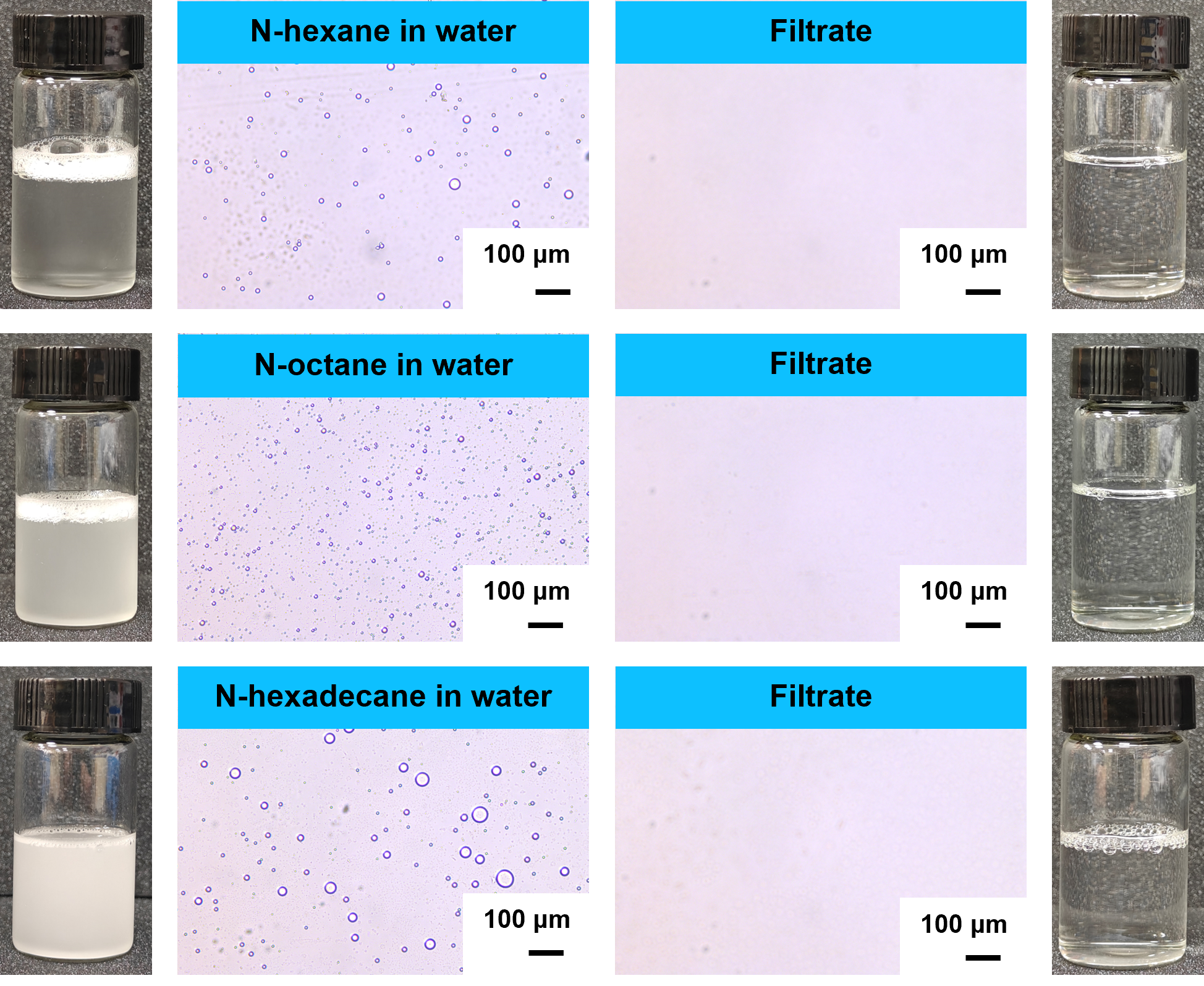


Figure S30. Appearance and microscopic images of O/W emulsion before and after membrane filtration. (The background color was globally normalized to improve the contrast between the oil and water phases)


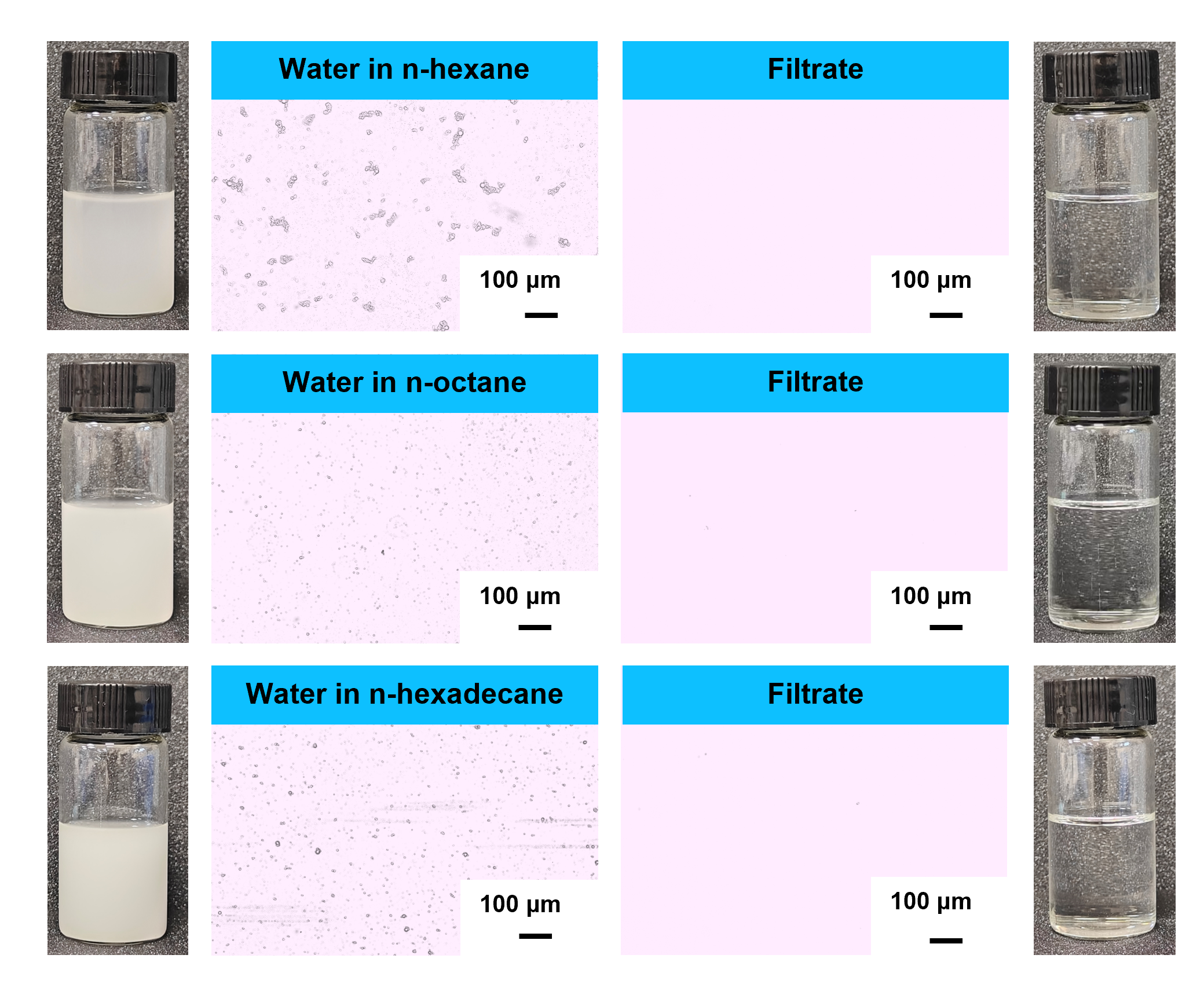


Figure S31. Appearance and microscopic images of W/O emulsion before and after membrane filtration. (The background color was globally normalized to improve the contrast between the oil and water phases)

Reference

[1] T. Lu, F. Chen, *J. Comput. Chem.* 2012, 33 (5), 580, <https://doi.org/10.1002/jcc.22885>.

[2] E. Virga, B. Bos, P. M. Biesheuvel, A. Nijmeijer, W. M. de Vos, *J. Colloid Interface Sci.* 2020, 571, 222, <https://doi.org/10.1016/j.jcis.2020.03.032>.
